# Genome-wide gene phylogeny of CIPK family in cassava and expression analysis of partial drought-induced genes

**DOI:** 10.3389/fpls.2015.00914

**Published:** 2015-10-30

**Authors:** Wei Hu, Zhiqiang Xia, Yan Yan, Zehong Ding, Weiwei Tie, Lianzhe Wang, Meiling Zou, Yunxie Wei, Cheng Lu, Xiaowan Hou, Wenquan Wang, Ming Peng

**Affiliations:** ^1^Key Laboratory of Biology and Genetic Resources of Tropical Crops, Institute of Tropical Bioscience and Biotechnology, Chinese Academy of Tropical Agricultural SciencesHaikou, China; ^2^College of Life Science and Engineering, Henan University of Urban ConstructionPingdingshan, China

**Keywords:** abiotic stress, cassava, CBL-interacting protein kinases, gene expression, genome-wide, identification

## Abstract

Cassava is an important food and potential biofuel crop that is tolerant to multiple abiotic stressors. The mechanisms underlying these tolerances are currently less known. CBL-interacting protein kinases (CIPKs) have been shown to play crucial roles in plant developmental processes, hormone signaling transduction, and in the response to abiotic stress. However, no data is currently available about the CPK family in cassava. In this study, a total of 25 *CIPK* genes were identified from cassava genome based on our previous genome sequencing data. Phylogenetic analysis suggested that 25 MeCIPKs could be classified into four subfamilies, which was supported by exon-intron organizations and the architectures of conserved protein motifs. Transcriptomic analysis of a wild subspecies and two cultivated varieties showed that most *MeCIPKs* had different expression patterns between wild subspecies and cultivatars in different tissues or in response to drought stress. Some orthologous genes involved in CIPK interaction networks were identified between Arabidopsis and cassava. The interaction networks and co-expression patterns of these orthologous genes revealed that the crucial pathways controlled by CIPK networks may be involved in the differential response to drought stress in different accessions of cassava. Nine *MeCIPK* genes were selected to investigate their transcriptional response to various stimuli and the results showed the comprehensive response of the tested *MeCIPK* genes to osmotic, salt, cold, oxidative stressors, and ABA signaling. The identification and expression analysis of CIPK family suggested that *CIPK* genes are important components of development and multiple signal transduction pathways in cassava. The findings of this study will help lay a foundation for the functional characterization of the *CIPK* gene family and provide an improved understanding of abiotic stress responses and signaling transduction in cassava.

## Introduction

Calcium signaling is involved in multiple physiological and developmental processes. Calcium, an important second messenger, regulates various signal transduction pathways (Kolukisaoglu et al., 2004; Kanwar et al., 2014). Intracellular calcium levels are modulated when plants respond to light, pathogens, abiotic stresses, and hormones and undergo physiological processes, such as root hair elongation, guard cell regulation, and pollen tube growth (Evans et al., 2001; Harper, 2001; Knight and Knight, 2001; Kolukisaoglu et al., 2004). Calcium signals are sensed and decoded by Ca^2+^ sensors, including calmodulins (CaMs), calmodulin-like proteins (CMLs), calcineurin B-like proteins (CBLs), and calcium-dependent protein kinases (CDPKs; Kudla et al., 2010; Zhang et al., 2014).

CBL-interacting protein kinases (CIPKs) can specifically target CBLs to transduce the perceived calcium signal, belonging to the Ca^2+^-mediated CBL-CIPK network, in response to various stimuli (Deng et al., 2013; Zhang et al., 2014). CIPK proteins are composed of an N-terminal kinase domain and a C-terminal regulatory domain. In the C-terminal regulatory domain, the conserved NAF or FISL motif is responsible for interacting with Ca^2+^-bound CBLs, leading to the activation of the targeting CIPKs (Albrecht et al., 2001; Guo et al., 2001). In addition, the protein-phosphatase interaction (PPI) domain within the C-terminus of CIPKs can also target specific members of the protein phosphatase 2C (PP2C; Ohta et al., 2003).

To date, genome-wide analysis has identified 26 CIPKs in Arabidopsis (Kolukisaoglu et al., 2004), 34 CIPKs in rice (Kanwar et al., 2014; Zhang et al., 2014), 43 CIPKs in maize (Chen et al., 2011), and 27 CIPKs in poplar (Yu et al., 2007). Phylogenetic analysis of CIPKs from various species, including algae, moss, spikemoss, monocots, and eudicots reveals that most CIPKs are classified into intron-rich clade and intron-less clade, whereas all the CIPKs in algae, moss and spikemoss are grouped into intron-rich clade (Ye et al., 2013). It was proposed that the rates of intron creation are higher during earlier periods of plant evolution (Roy and Penny, 2007). These evidences indicate that the generation of CIPKs in intron-less clade of seed plants occurs after seed plants have been separated from the basal vascular plants. Additionally, analysis of the CIPKs expansion suggests that segmental duplications contribute to the expansion of CIPKs in both intron-rich and intron-less clades, while tandem duplication events are found only in intron-less clade (Ye et al., 2013). Moreover, phylogenomic evidence shows that the expansion of CBL-CIPK network from a small module, likely a single CBL-CIPK pair, exists in the ancestor of modern plants and algae. The conserved NAF motif mediates CBL-CIPK physical interactions, which supports the interpretation of CBL and CIPK homologs as functionally linked network components (Kleist et al., 2014).

In plants, CIPKs have been demonstrated to affect Na^+^, K^+^, and ${\text{NO}}_{3}^{-}$ transport across the plasma membrane or tonoplast (Zhang et al., 2014). AtCBL4 (AtSOS3) can interact with AtCIPK24 (AtSOS2), transferring the kinase to the plasma membrane and activating the plasma membrane-localized Na^+^/H^+^antiporter AtNHX7 (SOS1) and vacuolar H^+^-ATPase to improve salt tolerance in roots (Qiu et al., 2002; Batelli et al., 2007; Kudla et al., 2010). AtCBL10 can also target CIPK24; this interaction is related to the vacuolar compartments and protecting shoots from salt stress (Quan et al., 2007). Some *CIPK* genes in other species have been reported to function similarly to *AtCIPK24* (*AtSOS2*) by increasing plants' tolerance to salt stress, such as *MdCIPK6L, MdSOS2*, and *ZmCIPK16* (Zhao et al., 2009; Hu et al., 2011; Wang et al., 2012). *HbCIPK2* and *SlSOS2* have also been found to function on K^+^ homeostasis (Huertas et al., 2012; Li et al., 2012). Moreover, the AtCBL1/CBL9-AtCIPK23 complex is involved in phosphorylating and activating the K^+^ channel AtAKT1, resulting in cellular K^+^ homeostasis (Xu et al., 2006). AtCIPK23 can also phosphorylate T101 of CHL1 to maintain a low-level primary response in response to low nitrate concentrations (Yu et al., 2014). Additionally, some CIPK family genes have been shown to be involved in other developmental processes and hormone signaling, including auxin and abscisic acid (ABA; Luan et al., 2009; Weinl and Kudla, 2009). AtCIPK26 plays an important role in ABA signaling in seed germination by interacting with ABI1, ABI2, and ABI5 (Lyzenga et al., 2013). AtCIPK6 has been identified to function on auxin transport, thus affecting root development and salt stress response (Tripathi et al., 2009). Collectively, these evidences have demonstrated that CIPKs play crucial roles in response to abiotic stress. However, the identities and functions of the CIPK family genes in cassava are still unknown.

Cassava (*Manihot esculenta*) can produce an acceptable yield under the adverse climatic and nutrient-poor soil, such as drought, low phosphorus and low nitrogen, thus making it an important food security crop (Xu et al., 2013); however, the mechanisms involved in cassava tolerant to abiotic stress are unknown. An understanding the molecular mechanisms of abiotic stress responses in cassava is necessary for genetic improvement of stress resistance for cassava and other crops. The availability of complete genome sequences will allow for identification of all genes, which will enable analyzing potential *cis*-elements and trans-factors, monitoring the expression profiles for all predicted genes, and investigating all the metabolic pathways and signaling transduction pathways, which are necessary to elucidate cellular and whole-plant processes (Hirayama and Shinozaki, 2010). Previously, we finished the whole genome sequencing of cassava wild ancestors and cultivated varieties (Wang et al., 2014), which has made it possible for the first time to perform genome-wide identification and analysis of gene family members in cassava.

Due to the importance of CIPKs in Ca^2+^-mediated network in response to various stimuli, the CIPK family was selected to perform genome-wide analysis in cassava. In the present study, we identified 25 *CIPK* genes and analyzed their phylogenetic relationship, gene structure, protein motifs, expression profiles in different tissues and in response to drought stress, interaction networks and co-expression patterns. This systematic analysis revealed that cassava CIPKs have the basic characteristics of CIPK family and may be important contributors of its strong tolerance to drought stress. Further expression analysis of *MeCIPKs* under the treatments of abiotic stress and ABA suggested that some CIPKs are involved in the transcriptional response of abiotic stress. Based on the systemical analysis of cassava CIPKs and the functions of CIPKs in Arabidopsis, we speculate that some MeCIPK genes, including MeCIPK23, MeCIPK10, MeCIPK9, MeCIPK16, MeCIPK12, and MeCIPK15 may possibly function on the regulation of abiotic stress responses. Our results from cassava may provide a basis for future research on abiotic stress responses medicated by CIPKs in cassava and are more likely to be directly translatable to agriculturally important dicotyledons and tropical crops.

## Materials and methods

### Plant materials and treatments

Wild subspecies (W14, *M. esculenta* ssp. *flabellifolia*), originally collected in Brazil, is an accession of the wild cassava subspecies and is the nearest ancestor of cultivated cassava, a middle type between wild ancestor species, *M. esculenta* ssp. *flabellifolia* and modern cultivated species. It has the characteristics of low photosynthesis rate, tuber root yield, and starch content in root tubers, but strong tolerance to drought stress (Wang et al., 2014). KU50 is a representative cultivar of the cultivated cassava, *M. esculenta*. It was bred by Kasas University in Thailand and has been extensively used in commercial plantations in East Asia (Wang et al., 2014). It has high root yield, high starch content in root tubers, and vigorous plant growth with wide adaptability to unfavorable environmental conditions (Utsumi et al., 2012). Argentina 7 (Arg7), adapted to geographical high-latitude region of Argentina, is a variety containing elite agronomic traits, including a certain level of growth under moderate drought stress (Zhao et al., 2014). South China 124 (SC124), a widely planted cassava cultivar in China, can survive in prolonged severe drought stress (Zhao et al., 2014). All plants were grown in a non-air-conditioned glass house of the Chinese Academy of Tropical Agricultural Sciences (Haikou, China). The plants were grown from April to July 2013 during which time the temperature in the glass house ranged from 20 to 35°C. W14 and two cultivated varieties (KU50 and Arg7) were used to detect the transcriptional changes of cassava genes in different organs under normal growth conditions. Arg7, SC124, and W14 were selected to study the transcriptional response of cassava genes to drought stress. Stem segments with three nodes were cut from 8 month old cassava plants and inclined into pots with a mixture of soil and vermiculite (1:1) where they were regularly watered. For drought treatment, cassava seedlings were withheld water for 12 days. For osmotic and salt treatments, cassava seedlings were irrigated with 200 mM mannitol and 300 mM NaCl solution for 24 days, respectively. For oxidative treatment, cassava seedlings were irrigated with 10% H_2_O_2_ for 72 h. For ABA treatment, cassava seedlings were sprayed with 100 μM ABA for 72 h. For cold treatment, cassava seedlings were exposed to 4°C for 48 h, then returned to standard growth conditions for 14 days of recovery.

### Identification and phylogenetic analyses of the CIPK gene family in cassava

The whole protein sequence of cassava was downloaded from the cassava genome database (http://www.phytozome.net/cassava.php). The CIPK amino acid sequences of the Arabidopsis, rice and Populus were acquired from UniPort (http://www.uniprot.org/), RGAP (http://rice.plantbiology.msu.edu/) and NCBI databases (http://www.ncbi.nlm.nih.gov/), respectively. The local Hidden Markov Model-based searches (HMMER: http://hmmer.janelia.org/) in the protein sequence dataset were carried out with HMM profiles built from known CIPKs as queries to identify the predicted CIPK genes in cassava (Finn et al., 2011). Additionally, BLAST analyses with all the CIPKs of Arabidopsis as queries were adopted to identify the predicted CIPKs in cassava database. CIPK gene models of cassava were only accepted if they contained the conserved Pkinase domain and the NAF domain with the help of CDD with automatic mode (threshold 0.01 and maximum number of hits 500) (http://www.ncbi.nlm.nih.gov/cdd/) and PFAM (http://pfam.sanger.ac.uk/) databases using *E*-value 1.0, then the predicted CIPK sequences were subjected to multiple sequence alignments to detect their conserved domains with DNAMAN 6.0 software. Finally, a maximum likelihood phylogenetic tree was constructed on the basis of multiple alignments of the identified CIPKs from cassava with all the CIPKs from Arabidopsis, rice and Populus using Clustal X 2.0 and MEGA 5.0 with 1000 bootstrap replicates and pair-wise deletion option (Larkin et al., 2007; Tamura et al., 2011).

### Protein properties and sequence analyses

The relative molecular mass and isoelectric points of putative CIPK proteins were predicted using the ExPASy proteomics server (http://expasy.org/) database with automatic mode. Motif analyses of cassava CIPKs were performed by MEME software (http://meme.nbcr.net/meme/cgi-bin/meme.cgi). The optimum width of motifs ranged from 6 to 50, the maximum number of motifs was 18, and the other parameter settings used were default values (Tao et al., 2014). The identified motifs were further annotated according to InterProScan (http://www.ebi.ac.uk/Tools/pfa/iprscan/). The genome sequences of MeCIPKs were retrieved from the cassava database and the gene structures were analyzed by the GSDS 2.0 (http://gsds.cbi.pku.edu.cn/) software with FASTA format. The 1500 bp sequences upstream of the coding sequences of *MeCIPK* genes were used to analyze *cis*-elements in their promoter regions using PlantCARE (http://bioinformatics.psb.ugent.be/webtools/plantcare/html/) databases. Those elements involved in abiotic stress response, including ABA-responsive element (ABRE), dehydration-responsive element (DRE), and low temperature-responsive element (LTRE), were subjected to further analysis (Shinwari et al., 1998; Narusaka et al., 2003). Specific interaction network with experimental evidences of each CIPK family members were constructed using STRING (http://string-db.org/) with option value >0.8, which identifies 18 high confidence interactive proteins in Arabidopsis. Then, the homologs of these interactive proteins in cassava were identified with reciprocal best BLASTP analysis and their expression patterns after drought treatment were retrieved from RNA-seq data sets.

### Transcriptomic analysis

Total RNA was extracted from stems (ST, 90 days after planting), leaves (LE, 90 days after planting), early storage roots (ESR, 90 days after planting), middle storage roots (MSR, 150 days after planting), and last storage roots (LSR, 210 days after planting) in Arg7, KU50, and W14 under normal growth conditions, and RNA was also collected from leaves and roots of Arg7, SC124, and W14 under normal or 12-days water withholding treatment. RNA-seq technique was employed to measure the global expression of cassava genes. Total RNA was extracted from different cassava tissues using a plant RNA extraction kit (TIANGEN) according to the manufacturer's instructions. Three microgram of total RNA from each sample was converted into cDNA using RevertAid First Strand cDNA Synthesis Kit (Fermentas). The cDNA libraries were constructed according to the protocols of Illumina, which were subsequently subjected to sequencing by Illumina GAII following Illumina RNA-seq protocol. A total of 813.90 million 51-bp raw reads was generated from the 24 samples. Adapter sequences were removed from raw sequence reads using FASTX-toolkit (version 0.0.13, http://hannonlab.cshl.edu/fastx_toolkit/). Sequence quality was examined using FastQC (http://www.bioinformatics.babraham.ac.uk/projects/fastqc/) and low quality sequences (including reads with unknown base pairs “N”) were removed, which produced 796.08 million clean reads. Clean reads were mapped to cassava reference genome (version 4.1) derived from the phytozome website (ftp://ftp.jgi-psf.org/pub/compgen/phytozome/v9.0/Mesculenta/) using Tophat v.2.0.10 (http://tophat.cbcb.umd.edu/) (Trapnell et al., 2009), and 89.6% reads were aligned. The resulting alignment files are provided as input for Cufflinks to generate transcriptome assemblies (Trapnell et al., 2012). Gene expression levels were calculated as FPKM according to the length of the gene and reads count mapped to this gene: FPKM = total exon fragments/[mapped reads (millions) × exon length (kb)]. DEGseq was applied to identify differentially expressed genes with a random sampling model based on the read count for each gene (Wang et al., 2010).

### Real-time quantitative polymerase chain reaction (qRT-PCR) analysis

Expression of *MeCIPK* genes in response to various abiotic stress and ABA were examined by qRT-PCR analysis using SYBR® Premix Ex Taq™ (TaKaRa) chemistry on a Stratagene Mx3000P (Stratagene, CA, USA) instrument. The 2-month-old Arg7 variety was treated with 300 mM mannitol, 200 mM NaCl, low temperature (4°C), 10 mM H_2_O_2_, and 100 μM ABA; then, the leaves of control and treated cassava plants were subsequently frozen in liquid nitrogen and stored at −80°C for extraction of total RNA and qRT-PCR assays. The relative expression of the tested genes with three replicates of each sample was assessed according to the 2^−Δ*ΔCt*^ method (Livak and Schmittgen, 2001). A series of primer and template dilutions were performed to acquire the optimal primer and template concentrations for amplifying the target genes prior to quantification experiments. Primers that had high specificity and efficiency on the basis of melting curve analysis and agarose gel electrophoresis were used to conduct quantification analysis (Table S11). Moreover, PCR products were sequenced to confirm the specificity of primer pairs. Amplification efficiencies of primer pairs ranged from 0.9 to 1.1. EF1 and TUB that were verified to be constitutive expression and suitable to be used as internal controls were used as reference genes to normalize transcriptional levels of target genes in this study (Salcedo et al., 2014).

## Results and discussion

### Identification and phylogenetic analysis of cassava *CIPK* genes

Both BLAST and Hidden Markov Model searches were carried out to extensively identify the MeCIPK proteins. A total of 25 non-redundant CIPKs (MeCIPK1-MeCIPK25) were identified from cassava genome. Conserved domain analysis confirmed that all the MeCIPKs identified harbor Pkinase and NAF domains, which are the basic characteristics of CIPK family. Moreover, multiple sequence alignment of CIPK proteins showed that all MeCIPKs identified in this study contain kinase domain in N-terminal and NAF/FISL domain in the C-terminal (Figure S1). As some genes contain alternative mRNA splicing, the longest protein for each gene was selected for further analyses. The 25 predicted MeCIPK proteins ranged from 362 to 499 amino acid residues and the relative molecular mass varied from 40.94 to 56.04 kDa (Table S1). All of the cDNAs of identified *MeCIPK* genes have been submitted to GenBank and the accession numbers of *CIPK* genes in cassava, Arabidopsis, rice, and Populus are shown in Table S2.

**Figure 1:**
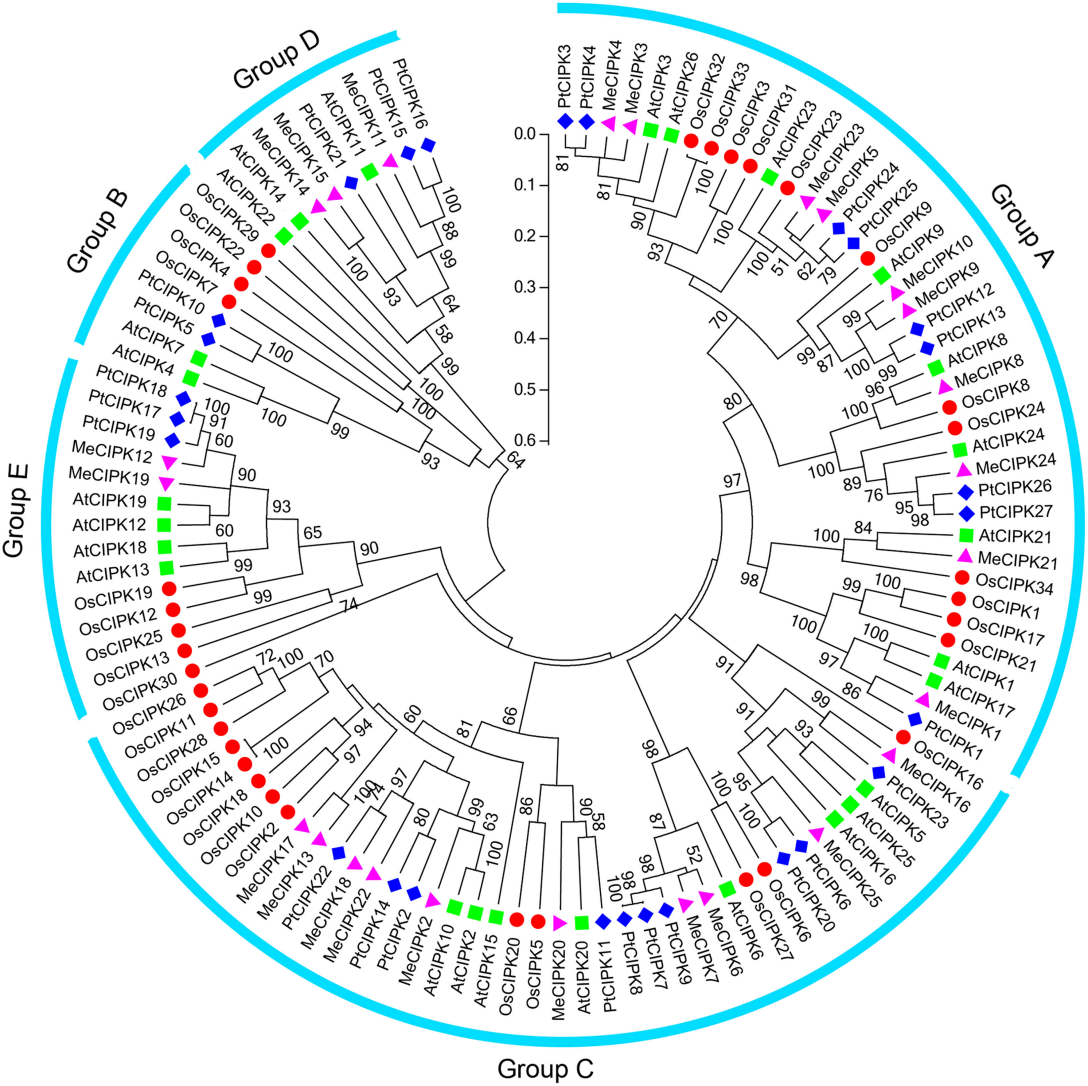
**Phylogenetic analysis of CIPK proteins in cassava, rice, Arabidopsis, and Populus**. A total of 25 CIPK proteins from cassava (triangle), 26 from Arabidopsis (square) and 34 from rice (round) and 27 Populus (diamond) were used to construct the maximum likelihood tree using ClustalX 2.0 and MEGA5 with 1000 bootstrap. Branches with less than 50% bootstrap support were collapsed. Five groups were labeled as A, B, C, D, and E.

To investigate the evolutionary relationship between cassava CIPK proteins and known CIPKs from other species, a maximum likelihood phylogenetic tree was constructed on the basis of the full amino acids of CIPK family proteins from cassava, rice, Arabidopsis, and Populus (Figure 1). The resulting dendrogram showed that all of the 112 CIPKs could be classified into five distinct groups (A to E) based on their sequence similarity, which is consistent with previous reports of CIPKs from Arabidopsis and Populus (Yu et al., 2007). The 25 MeCIPKs were assigned to four separate groups with high bootstrap values, together with 26 AtCIPKs, 34 OsCIPKs, and 27 PtCIPKs. Group A included MeCIPK1, -3, -4, -5, -8, -9, -10, -21, -23, -24, group C included MeCIPK2, -6, -7, -13, -16, -17, -18, -20, -22, -25, group D included MeCIPK11, -14, -15, Group E included MeCIPK12, -19, and no MeCIPK members were assigned to Group B. Similar phenomenon was also observed in the classification of CIPKs in canola, among which canola CIPKs were divided into four groups, together with CIPKs from Arabidopsis, rice, maize and other species (Zhang et al., 2014). As expected, CIPKs from cassava generally have closer relationships with the CIPK proteins from Arabidopsis and Populus than that from rice, which is accord with the current understanding of plant evolutionary history.

Phylogenetic analysis also identified some closely related orthologous CIPKs between cassava and Arabidopsis (MeCIPK24 and AtCIPK24, MeCIPK8 and AtCIPK8, MeCIPK21 and AtCIPK21 in Group A; MeCIPK20 and AtCIPK20 in Group C; MeCIPK11 and AtCIPK11 in Group D), suggesting that an ancestral set of CIPK genes existed prior to the divergence of cassava and Arabidopsis. AtCIPK24/AtSOS2, which shared a high degree of similarity with MeCIPK24, can interact with AtCBL4/AtSOS3 to function on the Na^+^/H^+^ antiporter, AtSOS1/AtNHX7, enhancing salt stress tolerance in roots of Arabidopsis (Kudla et al., 2010). AtCIPK8, the orthologs of MeCIPK8, is involved in the regulation of the low-affinity phase of the primary nitrate response (Hu et al., 2009). AtCIPK21 that showed high similarity with MeCIPK21 plays a positive role in osmotic and salt stress responses (Pandey et al., 2015). These evidences suggest the possible roles of CIPK genes in cassava.

### Gene structure, conserved motif, and *cis*-elements of cassava CIPKs

**Figure 2:**
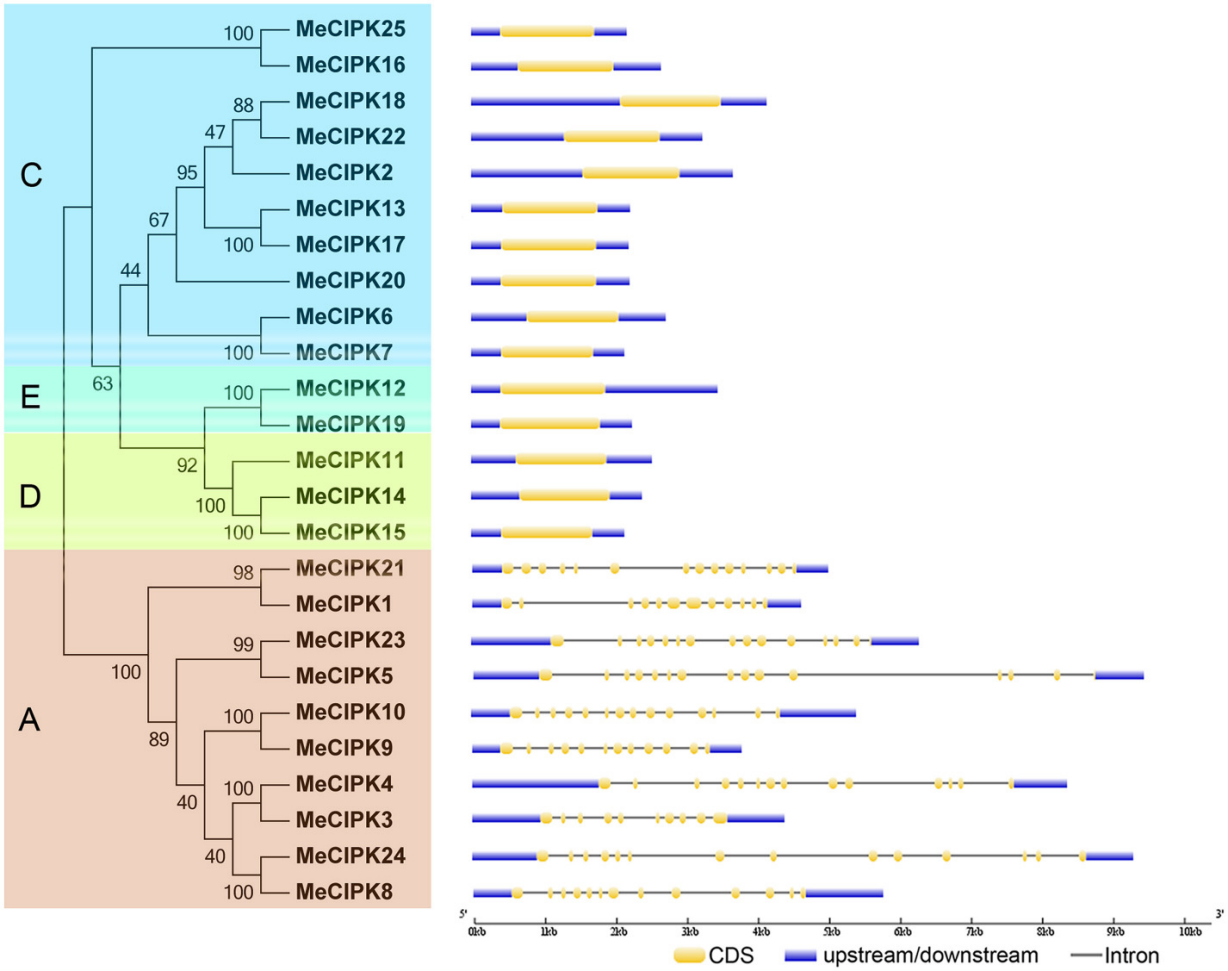
**The phylogenetic relationship and exon-intron structure analyses of CIPK family in cassava**. Exon-intron structure analysis was performed by online tool GSDS. Lengths of exons and introns of each MeCIPK gene were exhibited proportionally. A, C, D, and E indicated the classification of cassava CIPKs according to the phylogenetic relationship.

To better understand the structural features of cassava CIPKs, intron/exon distribution, conserved motifs, and *cis*-elements were analyzed according to their phylogenetic relationships. Phylogentic analysis indicated that 25 MeCIPK family members were divided into four groups (A, C, D, and E; Figure 2). Interestingly, the exon-rich *MeCIPK* genes containing 10–14 exons were clustered to group A, while the exon-poor members with only one exon were gathered to the other three groups (C, D, and E; Figure 2). The conserved exon numbers in each group supports their close evolutionary relationship and the classification of groups. In Arabidopsis, *CIPK* genes were also divided into exon-rich and exon-poor groups (Kolukisaoglu et al., 2004). Similar phenomenon were also observed for currently identified *CIPK* genes in both monocots and dicots species, such as rice, maize, and poplar (Yu et al., 2007; Kanwar et al., 2014), indicating the conserved features of the gene structure of the *CIPK* family. It was proposed that the rates of intron creation are higher during earlier periods of plant evolution (Roy and Penny, 2007). Additionally, according to Nuruzzaman et al. (2010), the rate of intron loss is faster than the rate of intron gain after segmental duplication. Thus, it is possible that the group A may represent the original genes of *CIPK* family.

**Figure 3:**
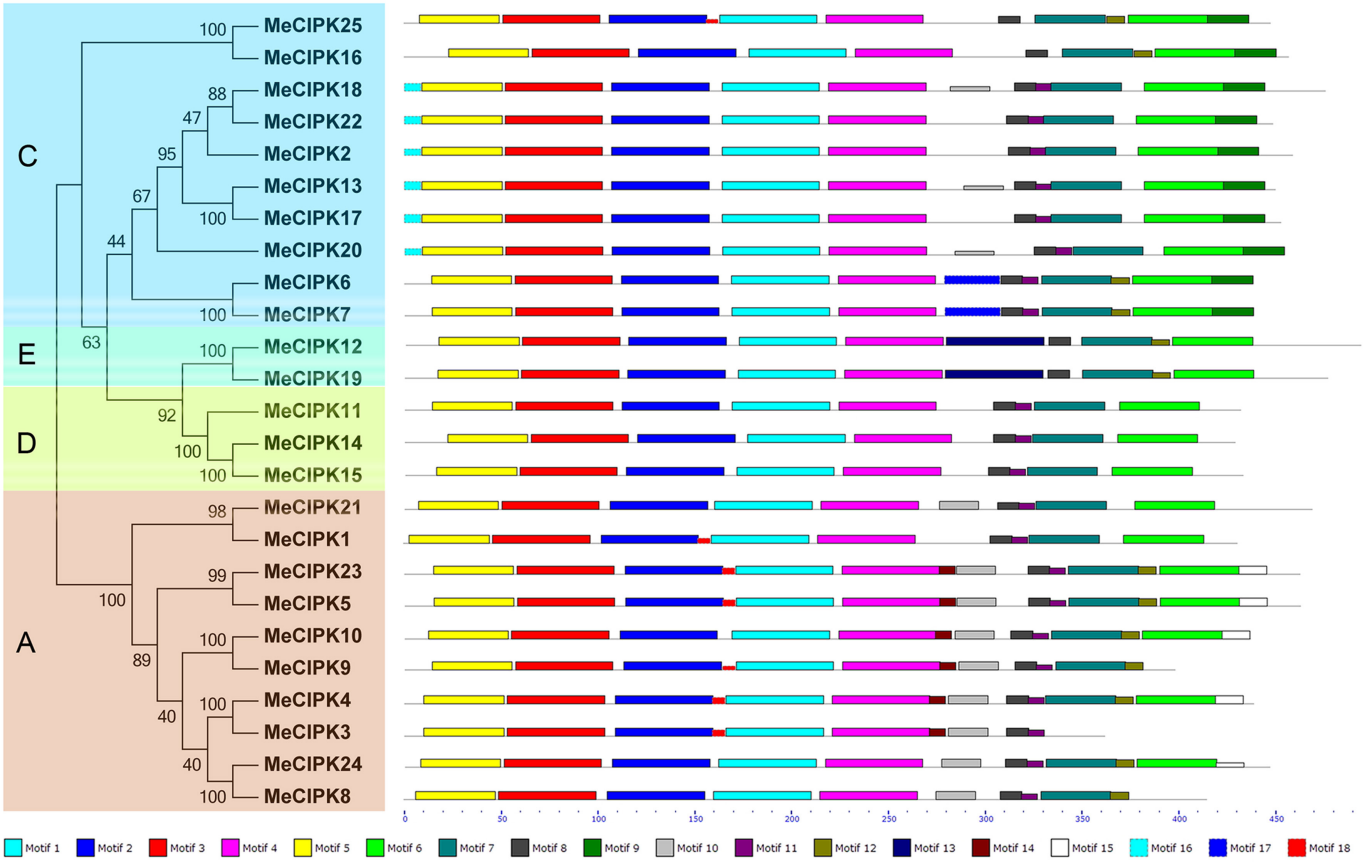
**Conserved motifs of cassava CIPK proteins according to the phylogenetic relationship**. All motifs were identified by MEME with the complete amino acid sequences of 25 CIPKs from cassava. Lengths of motifs of each MeCIPK protein were displayed proportionally. A, C, D, and E indicated the classification of cassava CIPKs according to the phylogenetic relationship.

The phylogenetic relationship and classification of cassava CIPKs were further supported by motif analysis (Figure 3; Figure S2; Table S3). Eighteen conserved motifs of cassava CIPKs were captured by motif analysis using MEME software and subsequent annotation with InterPro, in which 8 of the 18 captured motifs have functional annotations. Notably, all the cassava CIPK proteins, except for MeCIPK3, contain the motifs 1, 2, 3, 4, 5 that were annotated as Pkinase domain and the motifs 7, 8 that are closely related to NAF domain. Although MeCIPK3 lost the motif 7, it also contains Pkinase domain and NAF domain conferred by other motifs. CIPK proteins have the conserved characteristics of N-terminal catalytic kinase domain and the C-terminal regulatory domain that contains NAF motifs (Kanwar et al., 2014). Therefore, all the cassava CIPKs identified in this study has conserved features of the CIPK family, which is consistent with motif analysis of CIPKs in Arabidopsis (Figures S3, S4; Table S4). The conserved NAF motif mediates CBL-CIPK physical interactions, indicating that the identified CIPKs may be functionally linked in CBL-CIPK network (Kleist et al., 2014). Additionally, motif 11 annotated as the NAF domain was deficient in two paralogs (MeCIPK25, -16; MeCIPK12, -19). Furthermore, all the exon-rich MeCIPKs, except for MeCIPK1 in group A specially contain the motif 10, all of the group C MeCIPKs specially contain motif 9, the entire group C MeCIPKs, except for close related genes MeCIPK25/MeCIPK16 and MeCIPK6/MeCIPK7, specially show motif 16, and all of the group E MeCIPKs specially have motif 13. Generally, the majority of the conserved motifs existed in the same group, indicating that the classification of CIPK groups correlated with the identities of amino acid residue.

The *cis*-elements ABRE, DRE, and LTRE are involved in the abiotic stress response (Shinwari et al., 1998; Narusaka et al., 2003). These elements were identified in order to detect the possible response of cassava *CIPK* genes to abiotic stress. One Thousand five hundred base pair sequences upstream of the *MeCIPK* CDS region were selected to analyze those *cis*-elements. The results showed that 72% of the *MeCIPKs* have ABRE elements and 16% contain LTRE elements in their promoter region, indicating the transcriptional response of cassava *CIPK* family members to ABA and low temperature (Table S1).

### Expression patterns of cassava *CIPK* genes in different tissues

**Figure 4:**
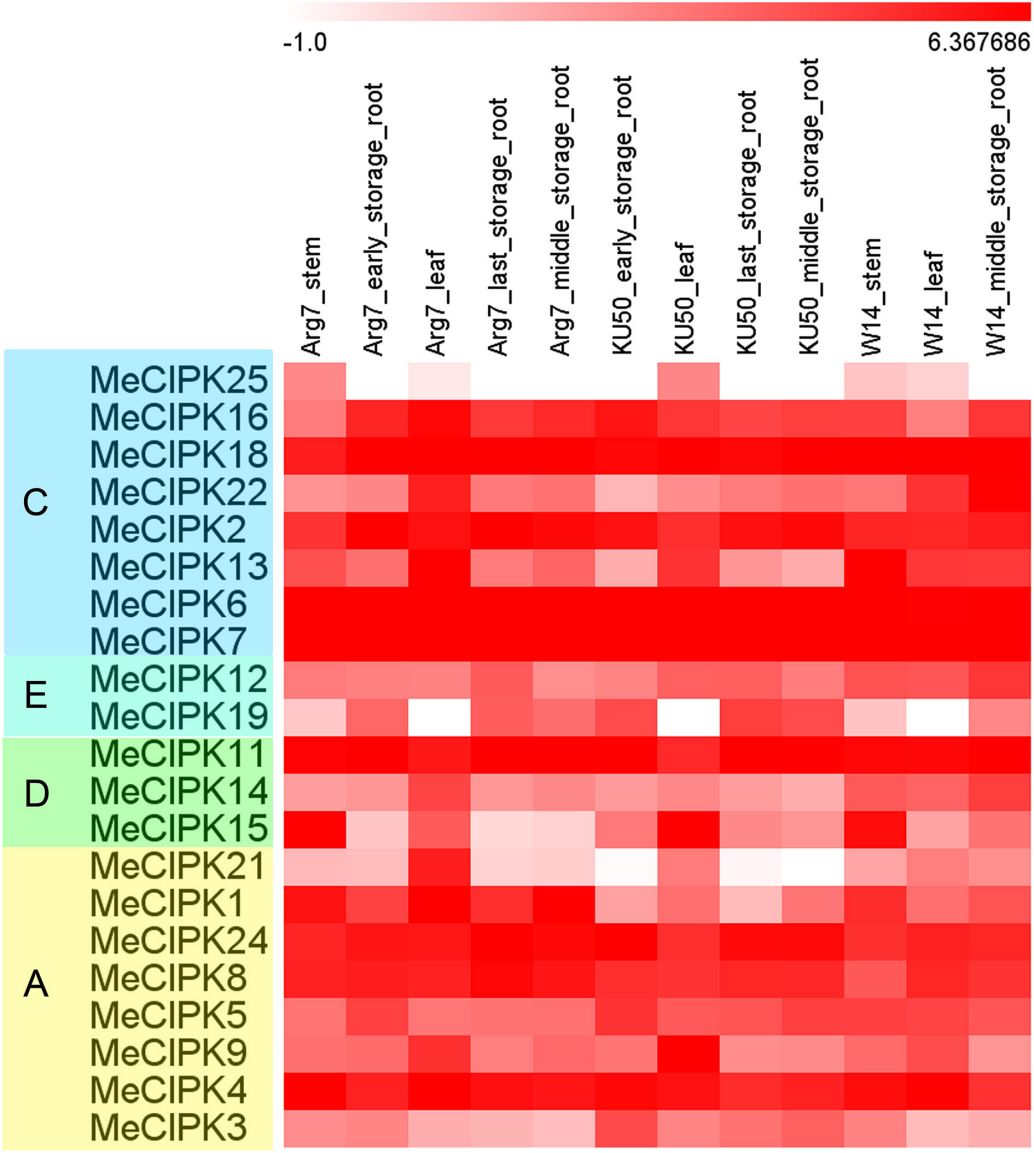
**Expression patterns of cassava CIPK genes in different tissues of three cassava accessions**. Log2 based FPKM value was used to create the heat map. The scale represents the relative signal intensity of FPKM values. A, C, D, and E indicated the classification of cassava CIPKs according to the phylogenetic relationship.

Several studies showed the involvement of *CIPK* family genes in different development stages (Tripathi et al., 2009; Kanwar et al., 2014). Thus, the holistic expression patterns of cassava *CIPKs* in different tissues are needed to provide more insight into their roles during plant growth and development. To detect the expression patterns of *CIPK* genes in different tissues, total RNA was extracted from stems (ST), leaves (LE), ESR, MSR, and LSR in a wild subspecies (W14) and two cultivated varieties (Arg7 and KU50). Transcriptiomic analysis revealed a transcript abundance of 21 *MeCIPK* genes in different tissues (Figure 4; Table S5). The expression of the other four *MeCIPK* genes *MeCIPK10, -17, -20, -23* was not detectable from the RNA-seq data. Heatmap representation of expression profile analysis suggested that the 21 *MeCIPK* members expressed in all the tested organs. For LE tissue, most *MeCIPK* genes showed higher expression levels in Arg7 than that in KU50 and W14. For ST tissue, most *MeCIPK* genes showed higher expression levels in W14 than that in Arg7. For MSR tissue, most *MeCIPK* genes showed higher expression levels in W14 than that in Arg7 and KU50. These results suggested that most of *MeCIPK* genes had differential expression in different accessions for a special tissue, which might contribute to the function diversity of different accessions for a special tissue.

*MeCIPK18, -6, -7, -11, -15, -1, -4* had high expression levels (value > 5) at ST tissue in both Arg7 and W14, *MeCIPK16, -18, -2, -6, -7, -11, -24, -8, -4* had high expression levels (value > 5) at ESR tissue in both Arg7 and KU50, *MeCIPK18, -6, -7, -11, -4, -2, -24* had high expression levels (value > 5) at LE tissue in Arg7, KU50, and W14, *MeCIPK18, -2, -6, -7, -11, -24, -8, -4* had high expression levels (value > 5) at LSR tissue in both Arg7 and KU50, and *MeCIPK18, -2, -6, -7, -11, -24, -4* had high expression levels (value > 5) at MSR tissue in Arg7, KU50, and W14. The strong expression of these *CIPK* genes for a special tissue in wild subspecies and cultivated varieties implied their key roles in tissue development or tissue functions.

Notably, *MeCIPK24, -8, -4* in group A, *MeCIPK6, -7*,-*16, -18, -2* in group C, and MeCIPK11 in group D have more transcript abundance (value > 5 in most tissues) than other *CIPK* genes. It is interesting to speculate that these *CIPK* genes with relative high transcripts in all the tested organs might play an important role in the development of cassava. In contrast, *MeCIPK25, -12, -19, -14, -21, -3* showed lower expression levels in different tissues (value < 3 in most tissues). Furthermore, some closely related genes exhibited similar expression profiles. For example, *MeCIPK6, -7* in group C have strong expression in various tissues tested (value > 5). *MeCIPK12, -19* and *MeCIPK14, -15* showed low transcript abundance (value < 3 in most tissues) in different tissues. However, some closely related genes showed different expression patterns, such as *MeCIPK18* and *MeCIPK22, MeCIPK21* and *MeCIPK1*, and *MeCIPK4* and *MeCIPK3*. Together, the tissue expression profiles of *CIPK* genes in different accessions will provide clues for further investigation of cassava development.

### Expression analysis of *MeCIPK* genes under drought treatment

**Figure 5:**
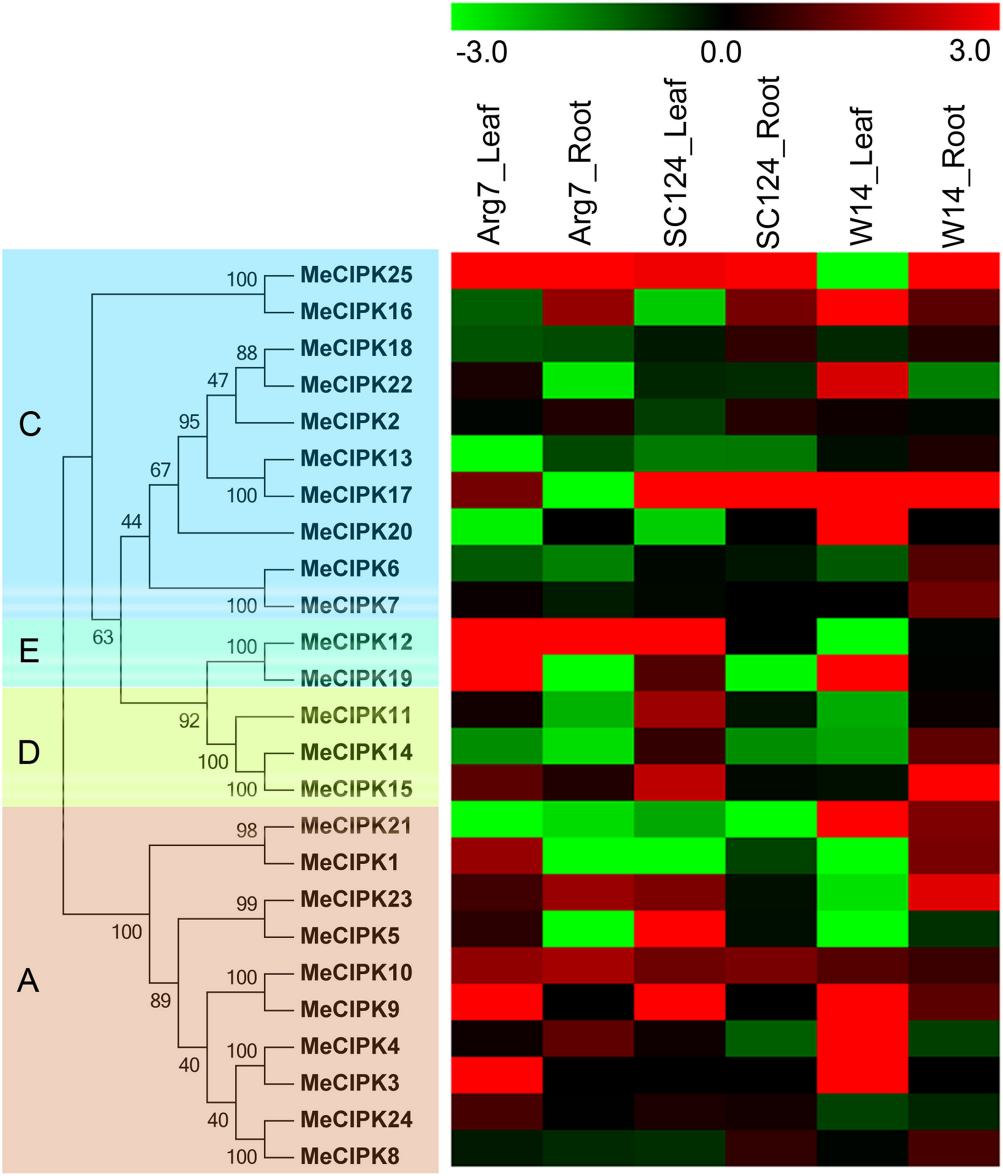
**Expression patterns of CIPK genes in leaves and roots of three cassava accessions after drought treatment**. Log2 based FPKM value was used to create the heat map. The scale represents the relative signal intensity of FPKM values. A, C, D, and E indicated the classification of cassava CIPKs according to the phylogenetic relationship.

*CIPK* family genes have been confirmed to play an important role in plants' response to drought or osmotic stress (Wang et al., 2012; He et al., 2013). Genome-wide expression analyses of *CIPK* genes in response to drought may lay a foundation for further understanding the mechanisms involved in strong tolerance of cassava. To investigate the transcriptional response of cassava *CIPK* genes to drought stress, water withholding was applied to a wild subspecies W14 and two cultivated varieties Arg7 and SC124. Total RNA was isolated from leaves and roots of control and drought-treated cassava plants. Global transcriptomic analysis showed transcriptional response of all the 25 *MeCIPK* genes after treatments compared to control plants (Figure 5; Table S6). In Arg7 variety, 44% and 24% of *MeCIPK* genes were transcriptionally induced by drought stress in leaves and roots, respectively. In SC124 variety, 48 and 28% of *MeCIPK* genes showed induction after drought treatment in leaves and roots, respectively. In W14 subspecies, 36 and 64% of *MeCIPK* genes were upregulated by drought stress in leaves and roots, respectively. These results suggested that the total number of *MeCIPK* family members induced by drought at transcriptional levels is more in W14 than that in Arg7 and SC124. W14 is a wild subspecies that shows stronger tolerance to drought than SC124 and Arg7, two varieties commonly cultivated in China and Southeast Asia, respectively (Wang et al., 2014). Moreover, the tolerance of these three accessions to drought stress was also confirmed before performing RNA-seq analysis (Figure S5). Accumulated evidence has demonstrated the function and transcriptional response of *CIPK* family genes in response to drought stress. Some *CIPK* genes, such as *AtCIPK6, OsCIPK12, OsCIPK23, MdCIPK6L, NtCIPK2, GhCIPK6*, were reported to be upregulated by drought and conferred tolerance to drought stress, indicating the positive role of *CIPK* family members to drought stress (Xiang et al., 2007; Yang et al., 2008; Tsou et al., 2012; Wang et al., 2012; He et al., 2013; Zhang et al., 2014). In Arabidopsis, expression of 4 *CIPK* genes (*AtCIPK6, -9, -11, -23*), accounting for 15%, were significantly induced by drought stress (Zhang et al., 2014). In rice, 12 *CIPK* genes (*OsCIPK1, -2, -5, -6, -14, -17, -19, -23, -24, -25, -31, -32*), accounting for 35%, were transcriptionally upregulated by drought treatment (Kanwar et al., 2014). In the present study, the high ratio of *CIPK* members (average 50% in roots and leaves) induced by drought at transcriptional levels in W14 subspecies might contribute to its strong drought tolerance.

Additionally, the number of *CIPK* genes transcriptionally upregulated by drought was greater in roots than that in leaves in W14, while less in roots than that in leaves in Arg7 and SC124. Cassava not only can form deep root systems (below 2 m soil depth) but also can penetrate into deeper soil layers, thus allowing the crop to absorb storage water of soil, although cassava has sparse fine root systems compared with other crops such as cereals and tropical grasses (Okogbenin et al., 2013). These results allow us to speculate that *MeCIPK* genes might mainly play a regulatory role in water uptake from soil by roots when cassava was subjected to drought stress in W14 subspecies. Generally, *MeCIPK* genes had similar expression patterns for leaves and roots tissues in Arg7 and SC124, which is different from W14. Most of *MeCIPK* genes showed upregulation in roots of W14, but downregulation in roots of SC124 and Arg7, and most of *MeCIPK* genes transcripts increased in leaves of W14, but decreased in leaves of SC124 and Arg7. The differential response of *CIPK* genes to drought in different accessions implied that the mechanisms driven by *CIPK* genes in response to drought are different between wild subspecies and cultivated varieties.

Notably, *MeCIPK23, MeCIPK10*, and *MeCIPK9* in group A, *MeCIPK25, MeCIPK16*, and *MeCIPK17* in group C, *MeCIPK15* in group D, and *MeCIPK12* in group E showed strong induction in the majority of the tested tissues. Additionally, some closely related genes generally have similar expression patterns, such as *MeCIPK6* and *MeCIPK7*; however, most of the paralogs did not show similar expression profiles under drought treatment. Taken together, the transcriptional response of *CIPK* genes to drought stress in wild subspecies and cultivated varieties suggested that cassava CIPKs may be important contributors of its strong tolerance to drought stress, which will be benefit for further investigation of the underlying mechanisms of strong drought tolerance in cassava.

### CIPK family interaction network and their co-expression in response to drought

**Figure 6:**
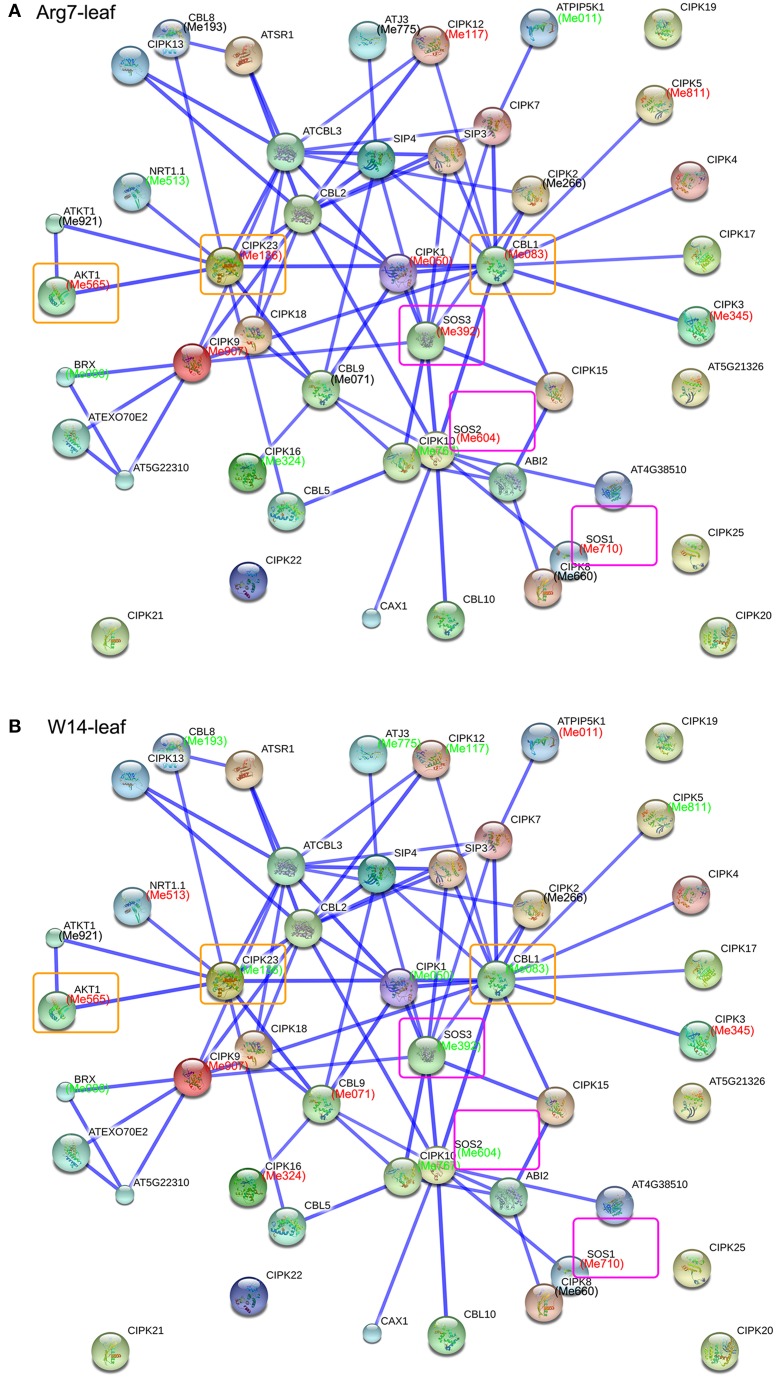
**Interaction network and co-expression analyses of CIPK genes in leaves of Arg7 (A) and W14 (B) and related genes in Arabidopsis**. The homologous genes of cassava are in parentheses. The genes marked with red font show upregulation based on 1.5 fold change. The genes marked with green font show downregulation based on 1.5 fold change. The genes involved in CBL4/SOS3-CIPK24/SOS2-SOS1/NHX7 and the CBL1/CBL9-CIPK23-AKT1 pathways are boxed with purple and yellow, respectively.

Genes implement their biological function and signaling transduction pathway typically through interaction networks. Investigation of potential interaction network associated with a gene family is benefit for understanding their functions (Tohge and Fernie, 2010). CIPKs have been demonstrated to interact with CBLs and several transporters to mediate multiple biological processes; thus, there is a need to identify possible interaction network of cassava CIPKs for better understanding their biological function based on the protein-protein interactions with experimental evidences in Arabidopsis. We constructed the networks of CIPKs using STRING software with option value >0.8, which identifies 20 high confidence interactive proteins involved in the CIPK family networks in Arabidopsis. These CIPK partners include CBLs involved in calcium ion binding, AKTs related to potassium transport, SOS1 related to sodium-hydrogen transport, ABI2 involved in protein dephosphorylation, CAX1 related to calcium ion transport, BRX involved in water transport, and other proteins with protein binding activity (Figure S6; Table S7). Previous functional analyses have clarified that these interactions participate in the regulation of plant ions transport, developmental processes, and hormone signaling and abiotic stress responses (Zhang et al., 2014). Further, the homologs of these interactive proteins were identified in cassava with reciprocal best BLASTP analysis and the expression profiles of each gene in response to drought were detected by RNA-seq data sets in Arg7 and W14 (Figure 6; Table S8). The expression patterns of those genes involved in the interaction networks were different in leaves of the Arg7 variety and W14 subspecies in cassava. The salt overly sensitive CBL4/SOS3-CIPK24/SOS2-SOS1/NHX7 and the CBL1/CBL9-CIPK23-AKT1 pathways are two crucial signaling pathways in the regulation of plant Na^+^ and K^+^ homeostasis, respectively (Qiu et al., 2002; Xu et al., 2006; Batelli et al., 2007; Kudla et al., 2010). In leaves of the Arg7 variety, the genes involved in both the two pathways show induction, implying their positive role in modulating cassava response to drought stress. However, this expression pattern was reversed in leaves of the W14 subspecies, among which the inhabitation of *CBL1-CIPK23* and *SOS3-SOS2* complexes resulted in induction of *AKT1* and *SOS1*, respectively. The differential response to drought stress in CIPK-mediated interaction networks implies that distinct mechanisms involved in cassava tolerant to drought stress are employed in wild subspecies and cultivated varieties.

### Expression profiles of *MeCIPK* genes in response to various stress and ABA treatments

**Figure 7:**
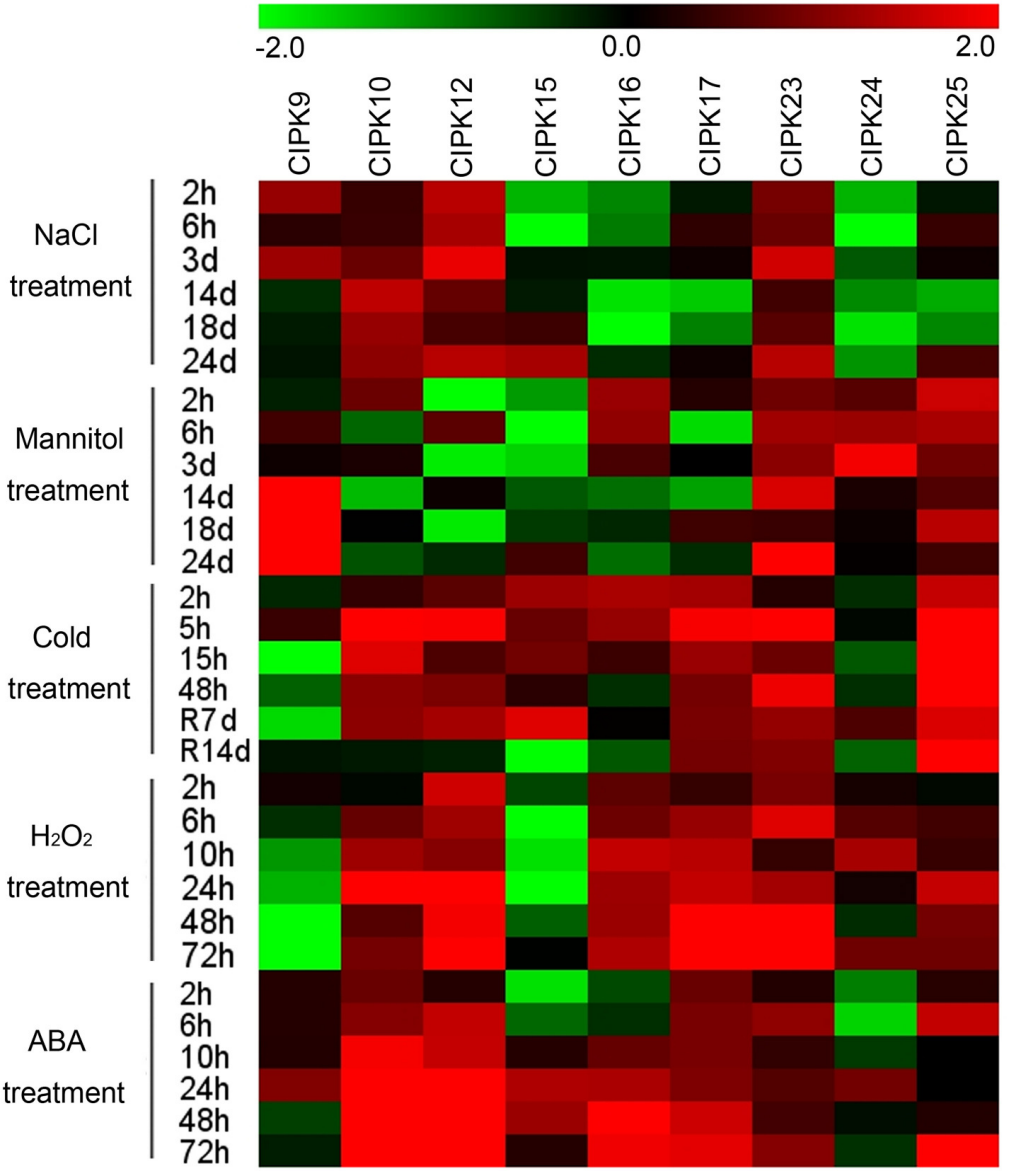
**Expression profiles of CIPK genes in leaves of cassava in response to osmotic, salt, cold, oxidative stresses, and ABA**. Log2 based value was used to create the heat map. The scale represents the relative signal intensity values.

Previous reports demonstrated the crucial roles of *CIPK* genes in various stress responses and in hormone signaling transduction (Quan et al., 2007; Weinl and Kudla, 2009), which motivated us to perform expression profile analyses of cassava *CIPK* genes under various abiotic stress and ABA treatments. Nine *MeCIPK* genes (*MeCIPK9, 10*, -*12, -15, -16, -17, -23, -24, -25*) distributed in different groups and transcriptionally induced in some tissues or in a special tissue by drought stress, based on our RNA-seq data in different cassava accessions, were selected to further examine their expression patterns in 2-month-old cassava leaves (Arg7) with the treatments of osmotic, salt, cold, H_2_O_2_, and ABA (Figure 7; Table S9). Under salt treatment, *MeCIPK9*, -*10*, -*12*, -*23* had increased transcripts. The expression of *MeCIPK15* was inhibited at early treated stages (2 and 6 h treatments), whereas *MeCIPK17* and *MeCIPK25* transcripts was inhibited at later treated stages (14 and 18 days). *MeCIPK16* and *MeCIPK24* expression were inhibited at all the treated time points (Figure 7; Figure S7). Under osmotic treatment, *MeCIPK16* and *MeCIPK24* showed upregulation at the 2 h, 6 h, and 3 day time-points, *MeCIPK23* and *MeCIPK25* showed induction at all the tested time-points, *MeCIPK9* expression was induced during 14–24 days treatment, *MeCIPK15* expression was inhibited during 2 h–18 days osmotic treatment, and *MeCIPK10, MeCIPK12*, and *MeCIPK17* expression did not display obvious trend during osmotic treatment (Figure 7; Figure S8). Under cold treatment, all the tested *MeCIPK* genes, except for *MeCIPK9* and *MeCIPK24*, were obviously upregulated during 2–48 h treatments. After 7 days recovery, *MeCIPK10*, -*12*, -*15*, -*17*, -*23*, -*25* also had increased transcripts. After 14 days recovery, *MeCIPK17*, -*23*, -*25* remained induction (Figure 7; Figure S9). Under H_2_O_2_ treatment, *MeCIPK10*, -*12*, -*16*, -*17*, -*23*, -*25* showed induction during 2–72 h treatments, *MeCIPK24* transcription was upregulated at 6, 10, and 72 h treatment, *MeCIPK9* transcripts decreased after 6–72 h treatment, and *MeCIPK15* expression decreased during 2–48 h treatments (Figure 7; Figure S10). Under ABA treatment, *MeCIPK10*, -*12*, -*17* expression were induced during 2–72 h treatments, *MeCIPK15* and *MeCIPK16* showed similar expression patterns with decreased transcripts at 2 and 6 h treatment and increased transcripts during 10–72 h treatment, the expression of *MeCIPK23* and *MeCIPK25* was upregulated at 6 h and 72 h treatment, and *MeCIPK9* and *MeCIPK24* expression was induced at 24 h treatment (Figure 7; Figure S11). Therefore, these *MeCIPKs* showed comprehensive response to abiotic stress and ABA signaling at transcriptional levels, indicating their possible roles in regulating cassava tolerance to abiotic stress.

To better understand the response of *CIPK* genes to abiotic stress and ABA, we compared the expression patterns of *CIPK* genes in cassava and Arabidopsis (Table S10). We focused only on Arabidopsis genes whose orthologs in cassava have been examined by qRT-PCR as suggested above. The expression profiles of *CIPKs* in Arabidopsis were reported by Zhang et al. (2014) according to microarray data. *AtCIPK9* (the orthologs of *MeCIPK9* and *MeCIPK10*) expression was induced by cold, salt and ABA treatments, *AtCIPK12* (the orthologs of *MeCIPK12* and *MeCIPK19*) transcription was induced by cold and ABA treatments, *AtCIPK14* (the orthologs of *MeCIPK14* and *MeCIPK15*) transcripts were induced by drought, salt, and ABA treatments, *AtCIPK16* (the orthologs of *MeCIPK16* and *MeCIPK25*) expression was upregulated by salt and ABA treatments, *AtCIPK24* (the orthologs of *MeCIPK24*) transcription did not show obvious changes after various treatments, and *AtCIPK23* (the orthologs of *MeCIPK23* and *MeCIPK5*) transcription was induced by oxidative stress. From these results, we found that some orthologs were both induced by special treatments, such as *MeCIPK10* and *AtCIPK9* induced by cold, salt and ABA treatments and *MeCIPK15* and *AtCIPK14* induced by drought treatment. Previously, *AtCIPK9* was reported to positively regulate resistance to low potassium stress and *AtCIPK14* played a positive role in the regulation of ABA signaling and salt stress response (Pandey et al., 2007; Qin et al., 2008). Here, we provide evidences that *AtCIPK9/MeCIPK10* may participate in modulating cold and salt responses and that *AtCIPK14/MeCIPK15* may be involved in the drought stress response.

Generally, *CIPK* genes positively respond to these stressors and ABA treatments more comprehensively in cassava than in Arabidopsis (Figure 7; Table S10). For example, *MeCIPK16* expression was induced by mannitol, cold, H_2_O_2_, and ABA treatments, whereas *AtCIPK16* transcription was induced by ABA and salt stress, *MeCIPK23* transcripts were upregulated by all the tested treatments, while *AtCIPK23* expression was only upregulated by oxidative treatment. *AtCIPK16* transcripts was shown to be upregulated after salt treatment and overexpression of *AtCIPK16* leads to plants with significant reduction in shoot Na^+^ and greater salinity tolerance (Roy et al., 2013). *AtCIPK23* is known as a crucial positive regulator of plants K^+^ uptake under low-K^+^ conditions (Xu et al., 2006). The comprehensive response of the *CIPK* genes to various abiotic stress and ABA indicated cassava *CIPKs* may be convergence points of different signaling pathways and may contribute to genetic improvement of cassava tolerance to abiotic stress.

## Conclusions

In this study, we conducted a genome-wide survey of the CIPK family in cassava. *In silico* analysis of the cassava genome database identified 25 *CIPK* genes, supported by conserved domain and multiple sequence alignment analyses. Phylogenetic analyses of CIPKs from cassava, Arabidopsis, and rice indicated that these CIPKs could be divided into four subfamilies. This classification was further supported by gene structure and motif analyses, with each group sharing common features of exon-intron and protein motifs. Transcriptomic analysis of a wild subspecies and two cultivators suggested that most of cassava *CIPK* genes had differential expression of different accessions for a special tissue, which might contribute to the function diversity of different accessions. Further transcriptomic analysis of different cassava accessions in response to drought revealed that the high ratio of *CIPK* members induced by drought in W14 subspecies might contribute to its strong tolerance to drought. Interaction network and co-expression analyses of cassava CIPKs demonstrated that the crucial pathways controlled by CIPK networks may be involved in differential responses to drought stress in different tissue or different accessions of cassava. Expression analysis of cassava *CIPK* genes responding to various abiotic stress and ABA treatments showed the comprehensive response of the *CIPK* genes to various abiotic stressors and ABA, implying that *CIPKs* may be the convergence points of different signaling pathways. Overall, our genome-wide identification and expression analysis of CIPK family provide a solid foundation for further functional dissection of CIPK family in cassava.

## Author contributions

MP and WW conceived the study. WH, ZX, YY, ZD, WT, LW, MZ, YW, CL, XH performed the experiments and carried out the analysis. WH and ZX designed the experiments and wrote the manuscript. All authors read and approved the final manuscript.

### Conflict of interest statement

The authors declare that the research was conducted in the absence of any commercial or financial relationships that could be construed as a potential conflict of interest.

## Supplementary material

Table S1 **Characteristics of CIPKs in cassava**.

Table S2 **The accession numbers of CIPKs in cassava, Arabidopsis, and rice**.

Table S3 **Conserved amino acid motifs and annotation of MeCIPKs**.

Table S4 **Conserved amino acid motifs and annotation of AtCIPKs**.

Table S5 **The expression profiles (log2-based values) of the cassava CIPK genes in different tissues**.

Table S6 **The expression profiles (log2-based values) of the cassava CIPK genes after drought treatment**.

Table S7 **Annotation summary of genes involved in the CIPK family interaction network**.

Table S8 **Expression patterns of the genes involved in CIPK-mediate interaction networks responding to drought in cassava**.

Table S9 **The expression profiles (log2-based values) of the cassava CIPKgenes after various abiotic stress and ABA treatments**.

Table S10 **Comparison the expression patterns of CIPK genes in cassava and Arabidopsis**. The orthologous genes in cassava and Arabidopsis were indicated with same color.

Table S11 **Primers used in qRT-PCR analysis**.

Table S12 **The accession number of transcriptomic data in NCBI**.

Figure S1 **Multiple sequence alignment of CIPK amino acid sequences in cassava**.

Figure S2 **Motifs of CIPKs in cassava**.

Figure S3 **Motifs of CIPKs in Arabidopsis**.

Figure S4 **Conserved motifs of cassava CIPK proteins according to the phylogenetic relationship**.

Figure S5 **Photos of different accessions of cassava after 12 days drought treatment**.

Figure S6 **Interaction network of AtCIPKs by STRING**.

Figure S7 **Expression profiles of CIPK genes in leaves of cassava in response to salt stress**. The relative expression levels of each gene are presented as the mean fold changes between treated and control samples at each time point. NTC indicates no treatment controls (mean value = 1). Data are means ± SD of *n* = 3 biological replicates.

Figure S8 **Expression profiles of CIPK genes in leaves of cassava in response to osmotic stress**. The relative expression levels of each gene are presented as the mean fold changes between treated and control samples at each time point. NTC indicates no treatment controls (mean value = 1). Data are means ± SD of *n* = 3 biological replicates.

Figure S9 **Expression profiles of CIPK genes in leaves of cassava in response to cold stress**. R7d and R14d indicate 7 days and 14 days recovery, respectively. The relative expression levels of each gene are presented as the mean fold changes between treated and control samples at each time point. NTC indicates no treatment controls (mean value = 1). Data are means ± SD of *n* = 3 biological replicates.

Figure S10 **Expression profiles of CIPK genes in leaves of cassava in response to oxidative stress**. The relative expression levels of each gene are presented as the mean fold changes between treated and control samples at each time point. NTC indicates no treatment controls (mean value = 1). Data are means ± SD of *n* = 3 biological replicates.

Figure S11 **Expression profiles of CIPK genes in leaves of cassava in response to ABA**. The relative expression levels of each gene are presented as the mean fold changes between treated and control samples at each time point. NTC indicates no treatment controls (mean value = 1). Data are means ± SD of *n* = 3 biological replicates.

### Availability of supporting data

The cassava CIPK genes identified in this study was submitted to GenBank (accession number: KP675718-KP675742). The transcriptomic data was submitted to NCBI and the accession number was listed in Table S12.

## References

[B1] Albrecht V. Ritz O. Linder S. Harter K. Kudla J. (2001). The NAF domain defines a novel protein-protein interaction module conserved in Ca^2+^-regulated kinases. EMBO J. 20, 1051–1063. 10.1093/emboj/20.5.1051 11230129 PMC145464

[B2] Batelli G. Verslues P. E. Agius F. Qiu Q. Fujii H. Pan S.. (2007). SOS2 promotes salt tolerance in part by interacting with the vacuolar H^+^-ATPase and upregulating its transport activity. Mol. Cell. Biol. 27, 7781–7790. 10.1128/MCB.00430-07 17875927 PMC2169139

[B3] Chen X. Gu Z. Xin D. Hao L. Liu C. Huang J.. (2011). Identification and characterization of putative CIPK genes in maize. J. Genet. Genomics 38, 77–87. 10.1016/j.jcg.2011.01.005 21356527

[B4] Deng X. Hu W. Wei S. Zhou S. Zhang F. Han J.. (2013). TaCIPK29, a CBL-interacting protein kinase gene from wheat, confers salt stress tolerance in transgenic tobacco. PLoS ONE 8:e69881. 10.1371/journal.pone.0069881 23922838 PMC3726728

[B5] Evans N. H. McAinsh M. R. Hetherington A. M. (2001). Calcium oscillations in higher plants. Curr. Opin. Plant Biol. 4, 415–420. 10.1016/S1369-5266(00)00194-1 11597499

[B6] Finn R. D. Clements J. Eddy S. R. (2011). HMMER web server: interactive sequence similarity searching. Nucleic Acids Res. 39, w29–w37. 10.1093/nar/gkr367 21593126 PMC3125773

[B7] Guo Y. Halfter U. Ishitani M. Zhu J. K. (2001). Molecular characterization of functional domains in the protein kinase SOS2 that is required for plant salt tolerance. Plant Cell 13, 1383–1400. 10.1105/tpc.13.6.1383 11402167 PMC135579

[B8] Harper J. F. (2001). Dissecting calcium oscillators in plant cells. Trends Plant Sci. 6, 395–397. 10.1016/S1360-1385(01)02023-4 11544110

[B9] He L. Yang X. Wang L. Zhu L. Zhou T. Deng J.. (2013). Molecular cloning and functional characterization of a novel cotton CBL-interacting protein kinase gene (GhCIPK6) reveals its involvement in multiple abiotic stress tolerance in transgenic plants. Biochem. Biophys. Res. Commun. 435, 209–215. 10.1016/j.bbrc.2013.04.080 23660187

[B10] Hirayama T. Shinozaki K. (2010). Research on plant abiotic stress responses in the post-genome era: past, present and future. Plant J. 61, 1041–1052. 10.1111/j.1365-313X.2010.04124.x 20409277

[B11] Hu D. G. Li M. Luo H. Dong Q. L. Yao Y. X. You C. X.. (2011). Molecular cloning and functional characterization of MdSOS2 reveals its involvement in salt tolerance in apple callus and Arabidopsis. Plant Cell Rep. 31, 713–722. 10.1007/s00299-011-1189-5 22108717

[B12] Hu H. C. Wang Y. Y. Tsay Y. F. (2009). AtCIPK8, a CBL-interacting protein kinase, regulates the low-affinity phase of the primary nitrate response. Plant J. 57, 264–278. 10.1111/j.1365-313X.2008.03685.x 18798873

[B13] Huertas R. Olías R. Eljakaoui Z. Gálvez F. J. Li J. De Morales P. A.. (2012). Overexpression of SlSOS2 (SlCIPK24) confers salt tolerance to transgenic tomato. Plant Cell Environ. 35, 1467–1482. 10.1111/j.1365-3040.2012.02504.x 22390672

[B14] Kanwar P. Sanyal S. K. Tokas I. Yadav A. K. Pandey A. Kapoor S.. (2014). Comprehensive structural, interaction and expression analysis of CBL and CIPK complement during abiotic stresses and development in rice. Cell Calcium 56, 81–95. 10.1016/j.ceca.2014.05.003 24970010

[B15] Kleist T. J. Spencley A. L. Luan S. (2014). Comparative phylogenomics of the CBL-CIPK calcium-decoding network in the moss *Physcomitrella*, Arabidopsis, and other green lineages. Front. Plant Sci. 5:187. 10.3389/fpls.2014.00187 24860579 PMC4030171

[B16] Knight H. Knight M. R. (2001). Abiotic stress signalling pathways: specificity and cross-talk. Trends Plant Sci. 6, 262–267. 10.1016/S1360-1385(01)01946-X 11378468

[B17] Kolukisaoglu U. Weinl S. Blazevic D. Batistic O. Kudla J. (2004). Calcium sensors and their interacting protein kinases: genomics of the Arabidopsis and rice CBL-CIPK signaling networks. Plant Physiol. 134, 43–58. 10.1104/pp.103.033068 14730064 PMC316286

[B18] Kudla J. Batistic O. Hashimoto K. (2010). Calcium signals: the lead currency of plant information processing. Plant Cell 22, 541–563. 10.1105/tpc.109.072686 20354197 PMC2861448

[B19] Larkin M. A. Blackshields G. Brown N. P. Chenna R. McGettigan P. A. McWilliam H.. (2007). Clustal W and Clustal X version 2.0. Bioinformatics 23, 2947–2948. 10.1093/bioinformatics/btm404 17846036

[B20] Li R. Zhang J. Wu G. Wang H. Chen Y. Wei J. (2012). HbCIPK2, a novel CBL-interacting protein kinase from halophyte *Hordeum brevisubulatum*, confers salt and osmotic stress tolerance. Plant Cell Environ. 35, 1582–1600. 10.1111/j.1365-3040.2012.02511.x 22458849

[B21] Livak K. J. Schmittgen T. D. (2001). Analysis of relative gene expression data using real-time Quantitative PCR and the 2^−Δ*ΔCt*^ method. Methods 25, 402–408. 10.1006/meth.2001.1262 11846609

[B22] Luan S. Lan W. Chul-Lee S. (2009). Potassium nutrition, sodium toxicity, and calcium signaling: connections through the CBL-CIPK network. Curr. Opin. Plant Biol. 12, 339–346. 10.1016/j.pbi.2009.05.003 19501014

[B23] Lyzenga W. J. Liu H. Schofield A. Muise-Hennessey A. Stone S. L. (2013). Arabidopsis CIPK26 interacts with KEG, components of the ABA signalling network and is degraded by the ubiquitin-proteasome system. J. Exp. Bot. 64, 2779–2791. 10.1093/jxb/ert123 23658427 PMC3697954

[B24] Narusaka Y. Nakashima K. Shinwari Z. K. Sakuma Y. Furihata T. Abe H.. (2003). Interaction between two cis-acting elements, ABRE and DRE, in ABA-dependent expression of Arabidopsis rd29A gene in response to dehydration and high-salinity stresses. Plant J. 34, 137–148. 10.1046/j.1365-313X.2003.01708.x 12694590

[B25] Nuruzzaman M. Manimekalai R. Sharoni A. M. Satoh K. Kondoh H. Ooka H.. (2010). Genome-wide analysis of NAC transcription factor family in rice. Gene 465, 30–44. 10.1016/j.gene.2010.06.008 20600702

[B26] Ohta M. Guo Y. Halfter U. Zhu J. K. (2003). A novel domain in the protein kinase SOS2 mediates interaction with the protein phosphatase 2C ABI2. Proc. Natl. Acad. Sci. U.S.A. 100, 11771–11776. 10.1073/pnas.2034853100 14504388 PMC208833

[B27] Okogbenin E. Setter T. L. Ferguson M. Mutegi R. Ceballos H. Olasanmi B.. (2013). Phenotypic approaches to drought in cassava: review. Front. Physiol. 4:93. 10.3389/fphys.2013.00093 23717282 PMC3650755

[B28] Pandey G. K. Cheong Y. H. Kim B. G. Grant J. J. Li L. Luan S. (2007). CIPK9: a calcium sensor-interacting protein kinase required for low-potassium tolerance in Arabidopsis. Cell Res. 17, 411–421. 10.1038/cr.2007.39 17486125

[B29] Pandey G. K. Kanwar P. Singh A. Steinhorst L. Pandey A. Yadav A. K.. (2015). CBL-interacting protein kinase, CIPK21, regulates osmotic and salt stress responses in Arabidopsis. Plant Physiol. 169, 780–792. 10.1104/pp.15.00623 26198257 PMC4577403

[B30] Qin Y. Li X. Guo M. Deng K. Lin J. Tang D.. (2008). Regulation of salt and ABA responses by CIPK14, a calcium sensor interacting protein kinase in Arabidopsis. Sci. China C. Life Sci. 51, 391–401. 10.1007/s11427-008-0059-z 18785584

[B31] Qiu Q. S. Guo Y. Dietrich M. A. Schumaker K. S. Zhu J. K. (2002). Regulation of SOS1, a plasma membrane Na^+^/H^+^ exchanger in Arabidopsis thaliana, by SOS2 and SOS3. Proc. Natl. Acad. Sci. U.S.A. 99, 8436–8441. 10.1073/pnas.122224699 12034882 PMC123085

[B32] Quan R. Lin H. Mendoza I. Zhang Y. Cao W. Yang Y.. (2007). SCABP8/CBL10, a putative calcium sensor, interacts with the protein kinase SOS2 to protect Arabidopsis shoots from salt stress. Plant Cell 19, 1415–1431. 10.1105/tpc.106.042291 17449811 PMC1913747

[B33] Roy S. J. Huang W. Wang X. J. Evrard A. Schmöckel S. M. Zafar Z. U.. (2013). A novel protein kinase involved in Na^+^ exclusion revealed from positional cloning. Plant Cell Environ. 36, 553–568. 10.1111/j.1365-3040.2012.02595.x 22897323

[B34] Roy S. W. Penny D. (2007). Patterns of intron loss and gain in plants: intron loss-dominated evolution and genome-wide comparison of *O. sativa* and *A. thaliana.* Mol. Biol. Evol. 24, 171–181. 10.1093/molbev/msl159 17065597

[B35] Salcedo A. Zambrana C. Siritunga D. (2014). Comparative expression analysis of reference genes in field-grown cassava. Tropical Plant Biol. 7, 53–64. 10.1007/s12042-014-9137-5

[B36] Shinwari Z. K. Nakashima K. Miura S. Kasuga M. Seki M. Yamaguchi-Shinozaki K.. (1998). An Arabidopsis gene family encoding DRE/CRT binding proteins involved in low-temperature-responsive gene expression. Biochem. Biophys. Res. Commun. 250, 161–170. 10.1006/bbrc.1998.9267 9735350

[B37] Tamura K. Peterson D. Peterson N. Stecher G. Nei M. Kumar S. (2011). MEGA5: molecular evolutionary genetics analysis using maximum likelihood, evolutionary distance, and maximum parsimony methods. Mol. Biol. Evol. 28, 2731–2739. 10.1093/molbev/msr121 21546353 PMC3203626

[B38] Tao P. Zhong X. Li B. Wang W. Yue Z. Lei J.. (2014). Genome-wide identification and characterization of aquaporin genes (AQPs) in Chinese cabbage (*Brassica rapa* ssp. *pekinensis*). Mol. Genet. Genomics 289, 1131–1145. 10.1007/s00438-014-0874-9 24972664

[B39] Tohge T. Fernie A. R. (2010). Combining genetic diversity, informatics and metabolomics to facilitate annotation of plant gene function. Nat. Protoc. 5, 1210–1227. 10.1038/nprot.2010.82 20539294

[B40] Trapnell C. Pachter L. Salzberg S. L. (2009). TopHat: discovering splice junctions with RNA-Seq. Bioinformatics 25, 1105–1111. 10.1093/bioinformatics/btp120 19289445 PMC2672628

[B41] Trapnell C. Roberts A. Goff L. Pertea G. Kim D. Kelley D. R.. (2012). Differential gene and transcript expression analysis of RNA-seq experiments with TopHat and Cufflinks. Nat. Protoc. 7, 562–578. 10.1038/nprot.2012.016 22383036 PMC3334321

[B42] Tripathi V. Parasuraman B. Laxmi A. Chattopadhyay D. (2009). CIPK6, a CBL-interacting protein kinase is required for development and salt tolerance in plants. Plant J. 58, 778–790. 10.1111/j.1365-313X.2009.03812.x 19187042

[B43] Tsou P. L. Lee S. Y. Allen N. S. Winter-Sederoff H. Robertson D. (2012). An ER-targeted calcium-binding peptide confers salt and drought tolerance mediated by CIPK6 in Arabidopsis. Planta 235, 539–552. 10.1007/s00425-011-1522-9 21971994

[B44] Utsumi Y. Tanaka M. Morosawa T. Kurotani A. Yoshida T. Mochida K.. (2012). Transcriptome analysis using a high-density oligomicroarray under drought stress in various genotypes of cassava: an important tropical crop. DNA Res. 19, 335–345. 10.1093/dnares/dss016 22619309 PMC3415295

[B45] Wang L. Feng Z. Wang X. Wang X. Zhang X. (2010). DEGseq: an R package for identifying differentially expressed genes from RNA-seq data. Bioinformatics 26, 136–138. 10.1093/bioinformatics/btp612 19855105

[B46] Wang R. K. Li L. L. Cao Z. H. Zhao Q. Li M. Zhang L. Y.. (2012). Molecular cloning and functional characterization of a novel apple MdCIPK6L gene reveals its involvement in multiple abiotic stress tolerance in transgenic plants. Plant Mol. Biol. 79, 123–135. 10.1007/s11103-012-9899-9 22382993

[B47] Wang W. Feng B. Xiao J. Xia Z. Zhou X. Li P.. (2014). Cassava genome from a wild ancestor to cultivated varieties. Nat. Commun. 5, 5110. 10.1038/ncomms6110 25300236 PMC4214410

[B48] Weinl S. Kudla J. (2009). The CBL-CIPK Ca^2+^-decoding signaling network: function and perspectives. New Phytol. 184, 517–528. 10.1111/j.1469-8137.2009.02938.x 19860013

[B49] Xiang Y. Huang Y. Xiong L. (2007). Characterization of stress-responsive CIPK genes in rice for stress tolerance improvement. Plant Physiol. 144, 1416–1428. 10.1104/pp.107.101295 17535819 PMC1914128

[B50] Xu J. Duan X. Yang J. Beeching J. R. Zhang P. (2013). Enhanced reactive oxygen species scavenging by overproduction of superoxide dismutase and catalase delays postharvest physiological deterioration of cassava storage roots. Plant Physiol. 161, 1517–1528. 10.1104/pp.112.212803 23344905 PMC3585613

[B51] Xu J. Li H. D. Chen L. Q. Wang Y. Liu L. L. He L.. (2006). A protein kinase, interacting with two calcineurin B-like proteins, regulates K^+^ transporter AKT1 in Arabidopsis. Cell 125, 1347–1360. 10.1016/j.cell.2006.06.011 16814720

[B52] Yang W. Kong Z. Omo-Ikerodah E. Xu W. Li Q. Xue Y. (2008). Calcineurin B-like interacting protein kinase OsCIPK23 functions in pollination and drought stress responses in rice (*Oryza sativa* L.). J. Genet. Genomics 35, 531–543. 10.1016/S1673-8527(08)60073-9 18804072

[B53] Ye C. Xia X. Yin W. (2013). Evolutionary analysis of CBL-interacting protein kinase gene family in plants. Plant Growth Regul. 71, 49–56. 10.1007/s10725-013-9808-5

[B54] Yu Q. An L. Li W. (2014). The CBL-CIPK network mediates different signaling pathways in plants. Plant Cell Rep. 33, 203–214. 10.1007/s00299-013-1507-1 24097244

[B55] Yu Y. Xia X. Yin W. Zhang H. (2007). Comparative genomic analysis of CIPK gene family in *Arabidopsis* and *Populus*. Plant Growth Regul. 52, 101–110. 10.1007/s10725-007-9165-3

[B56] Zhang H. Yang B. Liu W. Z. Li H. Wang L. Wang B.. (2014). Identification and characterization of CBL and CIPK gene families in canola (*Brassica napus* L.). BMC Plant Biol. 14:8. 10.1186/1471-2229-14-8 24397480 PMC3890537

[B57] Zhao J. Sun Z. Zheng J. Guo X. Dong Z. Huai J.. (2009). Cloning and characterization of a novel CBL-interacting protein kinase from maize. Plant Mol. Biol. 69, 661–674. 10.1007/s11103-008-9445-y 19105030

[B58] Zhao P. Liu P. Shao J. Li C. Wang B. Guo X.. (2014). Analysis of different strategies adapted by two cassava cultivars in response to drought stress: ensuring survival or continuing growth. J. Exp. Bot. 66, 1477–1488. 10.1093/jxb/eru507 25547914 PMC4438449

