# Supplementary material for: Genome-wide gene phylogeny of CIPK family in cassava and expression analysis of partial drought-induced genes
Source: Front Plant Sci. 2015 Oct 30;6:914. doi: 10.3389/fpls.2015.00914 (PMC4626571; doi:10.3389/fpls.2015.00914)
Supplement: Figure S1 — Multiple sequence alignment of CIPK amino acid sequences in cassava. [file Image1.PDF]

```
MeCIPK12        ---------MATKIHSNSN------SNQSPLLGRYEIGKLLGHGTFAKVYHARNVKTSES
MeCIPK19        ---------MAARIQANAN------SNQALLLGRYEIGKLLGHGTFAKVYLARNVKTNES
MeCIPK14        ---------MAE-DPSNPSPAIAEITPDINLFDKYELGKLLGCGAFAKVYHARNVVTGQS
MeCIPK15        ---------MADQEPTNP-------LPDVNLFEKYDLGKLLGSGAFAKVYHARNVRTGQS
MeCIPK11        ---------MPP-EIEQ--------VAENALFGKYELGKLLGCGAFAKVYHARNIRTGQS
MeCIPK6         ---------MAERSRDGHP---------TVLHGRYELGRMLGHGTFAKVYHARNLQTGKS
MeCIPK7         ---------MGERRRDGQS---------TLLHGRYELGRMLGHGTFAKVYLARNLQSGKS
MeCIPK18        --------------MENKG---------HVLMRKYEMGKLLGQGTFARVHHARNLQTGVS
MeCIPK22        --------------MENKG---------NVLMHKYEMGRLLGQGTFAKVHYARDIRTGTS
MeCIPK2         --------------MEHKP---------GILTQKYEIGRLLGQGTFAKVYYARSISTNQS
MeCIPK13        --------------MEHKG---------NVLMQRYDFGRLLGQGNFAKVYYARNLQTGQS
MeCIPK17        --------------MEHKG---------NVLMQRYDFGRLLGQGNFAKVYYARNLETGQS
MeCIPK20        --------------MEKKG---------AILMNRYELGRLLGQGTFAKVYHARNLQSGQS
MeCIPK16        MEVETERVAHKDCHINGAK---------HVLFGKYEMGKLLGQGTFAKVYKAKNLVTQER
MeCIPK25        ---------------MEER---------HVLFDKYEMGRLLGMGTFAKVYYGKNLATGES
MeCIPK23        ---------MASRGSAATG--------SRTRVGRYELGRTIGEGSFAKVKFARNTETGEN
MeCIPK5         ---------MKSRGSAATG--------NRTRVGKYELGRTLGEGTFAKVKFATNTETGEN
MeCIPK10        ----------MSVKAPS----------TRTRVGKYELGRTLGEGTFAKVKFAKNVETGEN
MeCIPK9         ----------MSVKVPSKQ--------TSTRVGKYELGRTLGEGTFAKVKFAKNVETGDS
MeCIPK4         --------------MSQPK--------IKRRVGKYEVGRTIGEGTFAKVKFARNSETGDP
MeCIPK3         --------------MSQPK--------IKRRVGKYEVGKTIGEGTFAKVKFARNSETGEP
MeCIPK24        ----------------MKK--------VTRKVGKYEVGRTIGEGTFAKVKFAQNMETGGS
MeCIPK8         ------------------M--------VVRKVGKYEIGRTIGEGTFAKVKFAQNTETGES
MeCIPK21        -----------------MG--------FVNNIGKYHLGKTIGEGTFAKVKLAVDSNNASY
MeCIPK1         -----------------------------MRLGKYELGRTLGEGNFGKVKLAKNIESGQP
                                                 :*..*: :* * *.:*  . .  .

MeCIPK12        VAIKVIDKEKILKGGLIAHIKREISILRRVRHPNIVQLFEVMATKAKIYFVMEYVRGGEL
MeCIPK19        VAIKVIDKEKILKGGLIAHIKREISILRRVRHPNIVQLFEVMATKAKIYFVMEYVRGGEL
MeCIPK14        VAIKAVSKQKVMKGGFIGQIKREISIMRRLHHPRVVKLLEVLATKTKIYFVMEFAKGGEL
MeCIPK15        VAIKAISKQKVVKGGFMGQVKREISIMRRLHHPNVVKLLEVLATKTKIYFVLEFAKGGEL
MeCIPK11        VAIKIINKKKIANTTLMSNIKREISIMRRLNHPHIVKLYEVLASKTKIYFVMEFVKGGEL
MeCIPK6         VAMKVVGKDKVIKVGMMEQIKREISVMKMVEHPNIVELHEVMASKSKIYFSMELVRGGEL
MeCIPK7         VAMKVVGKEKVIKVGMMEQIKREIAVMKMVKHPYIVDLHEVMASKSTIYFAMELVRGGEL
MeCIPK18        VAIKIIDKEKVLKVGMMEQIKREISVMRLIRHPNVVELYEVMATKSKIYFVMEYVKGGEL
MeCIPK22        VAIKIIDKEKIMKVGLMDQIKREISVMKLVRHPNIIQLYEVIATKTKIYYVLEYVKGGEL
MeCIPK2         VAIKVIDKEKILRAGLVDQIKREISVMRIVRHPNIVHLYEVMATKSKIYFVIEYCKGGEL
MeCIPK13        VAIKVIDKEKILKVGLINQIKREISVMKLVKHPNVLQLYEVMASKSKIYFAIEYAKGGEL
MeCIPK17        VAIKVIDKEKILKVGLINQIKREISIMKLVKHPNVLQLDEVMASKSKIYFVIEYAKGGEL
MeCIPK20        VAIKIINKEKILKGGLIDQIKREISVMRLVRHPNIVQLYEVMASKNKIYFAMEYVRGGEL
MeCIPK16        VAIKAIHKDHVKKKGLIEQIKREISVMHLVKHPNIVELKEVMATKSKVFFVMEYVRGGEL
MeCIPK25        VAIKVINKDQVKKEGMMEQIKREISVMRLVRHPNIVELKEVLATKSKIFFVMEYVRGGEL
MeCIPK23        VAIKILDKEKVLKHKMIGQIKREISTMKLIRHPNVIRMYEVMASKTKIYIVLEFVTGGEL
MeCIPK5         VAIKILDKENVLKHKMIVQIKHEISTMQLIRHPNVIRMYEVMACKTKIYIVLEFVTGGEL
MeCIPK10        VAIKIIDRDQILRLKMVEQLKREISTMKLIKHPNVIQIYEVMASKRKIYIVLEFVDGGEL
MeCIPK9         VAIKILDREQVLRLKMVEQLKREISTMKLIKHPNVIKIYEVMASKRKIYIVLEFVDGGEL
MeCIPK4         VALKILDKEKVLKHKMAEQIKREVATMKLIKHPNVVRLYEVMGSKTKIFIVLEFVTGGEL
MeCIPK3         VALKILDREKVLKHKMAEQIKREIATMKLIKHPNVVRLFEVMGSRTKIFIVLEFVTGGEL
MeCIPK24        VAMKVMAKSTILKHRMVDQIKREISIMKIVRHPNIVMLHEVLASRTKIYIILEFVTGGEL
MeCIPK8         VAMKVLDRSTIIKHKMVDQIKREISIMKLVRHPYVVRLHEVLASRTKIYIILEFITGGEL
MeCIPK21        VAVKIIDKQMVVESNLKYQVVREIKTMKILNHPNIVRIHEVIGTKRKIYIVMEYVSGGQL
MeCIPK1         FAVKILEKNRIIHLNITDQIKREIATLKLLKHHNVVRLYEVLASKSKIYMVLEYVNGGEL
                .*:* : :. : .  :  :: :*:  :: :.*  :: : **:. : .::  :*   **:*

MeCIPK12        FNKVA-KGRLKEEVARKYFQQLISAVGFCHARGVFHRDLKPENLLLDENGDLKVSDFGLS
MeCIPK19        FNKVA-KGRLKEEVARKYFQQLISAVSFCHARGVFHRDLKPENLLLDENGDLKVSDFGLS
MeCIPK14        FAKVA-KGRFSEDLSRKYFQQLITAVGYCHARGVFHRDLKPENLLLDENWDLKVSDFGLS
MeCIPK15        FTRVA-KGRFSEGLSRRYFQQLITAVEYCHSRGVFHRDLKLENLLLDDNWDLKVTDFGLS
MeCIPK11        FAKVA-KGRFSEDLSRKYFQQLISAVAYCHARGVFHRDLKPENLLLDESGNLKVSDFGLS
MeCIPK6         FAKIA-KGRLREDVARVYFQQLISAIGFCHSRGVYHRDLKPENLLLDEDGNLKVTDFGLS
MeCIPK7         FSRIS-KGRLREDVARVYFQQLISAVDFCHSRGVYHRDLKPENLLLDEDGNLKVTDFGLS
MeCIPK18        FNKVA-KGKLKEDIGRKYFQQLISAVDYCHSRGVSHRDLKPENLLLDENGNLKVSDFGLS
MeCIPK22        FNKVA-KSKLKEDVARKYFQQLISAVDFCHSRGVYHRDLKPENLLLDEDGNLKVSDFGLS
MeCIPK2         FNKVA-KGKLKEDVAHMYFHQLINAVDFCHSRGVYHRDIKPENLLLDENENLKISDFGLS
MeCIPK13        FKKVA-KGRLKEDVARNYFQQLISAVDFCHSRGVYHRDLKPENLLLDENGVLKVSDFGLS
MeCIPK17        FKKVA-KGRFNEDVARNYFHQLISAVDFCHSRGVYHRDLKPENLLLDENGVLKVSDFGLS
MeCIPK20        FNKVA-KGKLNEDVARKYFQQLIGAVDFCHSRGVYHRDIKPENLLLDENGNLKVSDFGLS
MeCIPK16        FAKLQ-QGKLKEDLARKYFQQLISAVDFCHSRGVSHRDLKPENLLLDENGDLKVSDFGLS
MeCIPK25        FAKVA-KGKLKEDVARKYFQQLTSAVDFCHSRGVCHRDLKPENLLLDENGDLKISDFGLS
MeCIPK23        FDKIASKGRLKEDEARKYFQQLINAVDYCHSRGVYHRDLKPENLLLDASGVLKVSDFGLS
MeCIPK5         FDKIASRGRLKEDEARKYFQQLINAVDYCHSRGVYHRDLKPENLLLDASGVLKVSDFGLS
MeCIPK10        FDKIARNGRLKEDEARRYFHQLIDAVDYCHSRGVSHRDLKPENLLLDSYGTLKVSDFGLS
MeCIPK9         FDKIATQGRLKEDEARRYFHQLINAVDYCHSRGVSHRDLKPENLLLDSLGVLKVSDFGLS
MeCIPK4         FDKIVNHGRMREDEARRYFQQLINAVDYCHSRGVYHRDLKPENLLLDANGNLKVSDFGLS
MeCIPK3         FDKIVNHGRMREDEARRYFQQLINAVDYCHSRGVYHRDLKPENLLLDACGNLKISDFGLS
MeCIPK24        FDKIVHQGRLSENEARRYFQQLIDAVAHCHSKGVYHRDLKPENLLLDSVGNLKVSDFGLS
MeCIPK8         FDKIVHHGRLSEAEARRYFQQLIDGVDYCHSKGVYHRDLKPENLLLDSQGNLKISDFGLS
MeCIPK21        SDKLSYSQKLKESEARKMFQQLIDAVDYCHNRGVFHRDLKPENLLLDSKGNMKVSDFGLS
MeCIPK1         FDRIASKGKLSEAQGRKLFQQLIDGVSYCHNKGVFHRDLKLENVLIDTKGNLKISDFGLS
                  ::    :: *  .:  *:**  .: .** :** ***:* **:*:*    :*::*****
```

```
                 Activation loop
MeCIPK12        AVS-DQIRQDGLFHTFCGTPAYVAPEVLARKGYDAAKVDIWSCGVILFVLMAGYLPFHDQ
MeCIPK19        AVS-DQIRQDGLFHTFCGTPAYVAPEVLARKGYDAAKVDIWSCGVILFVLMAGYLPFNDQ
MeCIPK14        AVN-DQIQSDGLLHTLCGTPAYVAPEILGKKGYDGAKADVWSCGVILYVLIAGYLPFNDT
MeCIPK15        AVK-DQIEPDGLLHTLCGTPAYVAPEILGKKGYDGAKVDVWSCGIILYVLIAGYLPFNDT
MeCIPK11        AVT-DQIRTDGLLHTLCGTPAYVAPEILAKKGYDGAKVDIWSCGVILFVLTAGYLPFNDP
MeCIPK6         AFT-DHLKQDGLLHTTCGTPAYVAPEVIGKKGYDGAKADLWSCGVILYVLLAGFLPFQDD
MeCIPK7         AFS-EHLKQDGLLHTTCGTPAYVAPEVIGKKGYDGAKADLWSCGVILYVLLAGFLPFQDD
MeCIPK18        ALA-ECKQQDGLLHTTCGTPAYVAPEVINRKGYDGSKADIWSCGVILYVLLAGYLPFHDT
MeCIPK22        ALA-ECTRQDGLLHTTCGSPAYVAPEVIKRKGYDGPKADIWSCGVILYVLLAGYLPFYDS
MeCIPK2         ALA-ESKRQDGLLHTTCGTPAYVAPEVINRKGYDGAKADIWSCGVVLFVLLAGYLPFHDS
MeCIPK13        ALV-ESKSQDGLLHTTCGTPAYVAPEVIKSKGYDGAKADIWSCGVILYVLLAGYLPFHDA
MeCIPK17        ALV-ESKAQDGMLHTTCGTPAYVAPEVISRKGYDGAKADIWSCGVILYVLLAGYLPFHDA
MeCIPK20        ALW-DSRKQDGLLHTTCGTPAYVAPEVITKNGYDGAKSDIWSCGVVLFVLLAGYLPFNHQ
MeCIPK16        ALP-EQHWNDGLLHTQCGTPAYVAPEVLRKKGYDGAKADIWSCGVILFVLLAGYLPFQNA
MeCIPK25        ALP-EHLRIDGLLHTQCGTPAYVAPEVLRKKGYDGSRADIWSCGVILYVLLAGFLPFRDE
MeCIPK23        ALP-QQVRDDGLLHTTCGTPNYVAPEVINNKGYDGAKADLWSCGVILFVLMAGYLPFEES
MeCIPK5         ALP-QQVREDGLLHTTCGTPNYVAPEVISNKGYDGAKADLWSCGVILFVLMAGYLPFEES
MeCIPK10        AVLSKQVQGDGLLHTACGTPNYVAPEVLKDKGYDGTGSDIWSCGVILYVLMAGYLPFDEP
MeCIPK9         AVLSNQIRGDGLLHTACGTPNYVAPEVLKDKGYDGTGSDVWSCGVILFVLMAGYLPFDEP
MeCIPK4         ALS-QQVRDDGLLHTTCGTPNYVAPEVLNDRGYDGATADMWSCGVILFVLLAGYLPFDDP
MeCIPK3         ALS-QQVRDDGLFHTACGTPNYVAPEVLNDRGYDGATADIWSCGVILFVLLAGYLPFDDD
MeCIPK24        ALP---QEGVGLLHTTCGTPNYVAPEVLSHQGYDGAAADVWSCGVILYVLMAGYLPFDEA
MeCIPK8         ALP---EQGVSLLRTTCGTPNYVAPEVLSHKGYNGAVADVWSCGVILYVLMAGYLPFDEL
MeCIPK21        ALR-KHG---DVLTTACGSPCYVAPELLANKGYDGAAADVWSCGVILFELLSGYLPFDDR
MeCIPK1         ALP-QHFRDDGLLHTTCGSPNYVAPEILSNRGYDGATSDIWSCGVILYVILTGYLPFDDR
                *.        .:: * **:* *****::  .**:..  *:****::*: : :*:*** .

MeCIPK12        NVMVMYKKIYKGEFRCPRWFSPELIRLLSKLLDTNPETRITIPEIMEN--KWFKRG-FKH
MeCIPK19        NIMAMYKKIYKGEFRCPRWFSPELVRLLWRLLDTNPETRGTMPQIMEN--KWFKKG-FKH
MeCIPK14        NLMVMYRKIYRGQFRFPKWTSPDLRRFLSRLLDANPETRITVDEILRD--PWFKQD-FKG
MeCIPK15        NIMSMYRKIYRGQFRFPKWTSPDLRRFLSRLLDTNPKTRITVDEIIQD--SWFKKD-YKG
MeCIPK11        NLMVMYKKIYKGEFRCPKWMSPDLKRFLSRVLDTNPQTRITVDEILKD--PWFKRGGLKE
MeCIPK6         NIVAMYRKIYRGDFKCPPWFSPEARRLITKLLDPNPSSRITISKIMDS--SWFKKSVPKT
MeCIPK7         NIVAMYRKIYRGDFKCPPWFSPEARRLITKLLDPNPSTRIAISKVMDS--SWFKKSMPKT
MeCIPK18        NLMEMYRKIGKAEFRFPNWFAPEVRKLLSKILDPKPSTRISMVKIMEN--SWFCKGLEPK
MeCIPK22        NLMEMYRRIAKADFKSPDWFAPEACRLLSKILDPNPRTRISIAEIIESPWYWYHRDLESQ
MeCIPK2         NLMEMYRKIGKADFKCPNWFPQEARRLLFKMLDPNPNTRISMDKIKES--SWFRKVFNPK
MeCIPK13        NLISLYRKITKAEYKCPNWFLPEVRKLVSKMLDPNPSTRISIEKIMEN--PWFRKGFNPK
MeCIPK17        NLISLYKKISKAEYKCPGWFSSEVRKLVSKMLDPNPRNRISIAKIMET--SWFKKGFNSK
MeCIPK20        NIMELYRKIIKGEFKCPTWINSGARKLISSILHPNPNSRIAIDDITSN--SWFKKGYKQI
MeCIPK16        NLMKMYVKIFKAEYEFPPWISPEARRLISKHLVVDPERRITIPHIRQN--PWFRRGLTTP
MeCIPK25        NVMKMYRQIFKAEFECPPWISTEAKRLISRLLVSDPERRITIPAITRV--PWFRKGFTRP
MeCIPK23        NLVALYKKIFKADFTCPPWFSSSAKKLIKRILDPNPSTRITIAEVIEN--EWFKKGYKPP
MeCIPK5         SLMALYKKIFKAEFTCPPWFSSSAKKLIKRILDPNPLTRITFAEVIAN--EWFKKGYKPP
MeCIPK10        SLMGLYKKICSADFTFPSCFSSGSRKLIKRILDPNPVTRITIPQILED--EWFKKGYKPP
MeCIPK9         SFMGLYGKICSADFTFPSWFSSGARKLIKRILDPNPVTRITIPEMLED--EWFKKGYKPP
MeCIPK4         NLMNLYKKISAAEFTCPPWLSFGAMKLITRILDPNPMTRITIPEILED--EWFKKDYKPP
MeCIPK3         NLINLYKKISAAEFTCPPWLSFGAMKLITRILDPNPVTRITISEILED--EWFKKDYKPP
MeCIPK24        DLPTLYRKINAAEYTCPFWFSPGAKALIDKILDPNPKTRIRIEGIRKN--PWFAKQYVPV
MeCIPK8         DLTTLYGKIENADFSCPSWFPVGAKSLIHRILDPKPETRITIEQIRND--EWFNKGYVPA
MeCIPK21        NLVVLYKKICAAEYTFPLWFTESQKKLISRILDPNPKRRITIAEMIED--EWFQTDYVPS
MeCIPK1         NLAVLYQKIFKGEAQIPKWLSPGAQNMIRRILDPNPLTRITVAGIEAD--EWFKQDYTPA
                ..  :* :*  .:   *         ::   *  .*  *  .  :      *:

MeCIPK12        IKFYIEDDKVFSVDVEGQQDDAGSSSDQSQCESEPEMETRRRITSLPRPASLNAFDIIS-
MeCIPK19        IKFFIEDDKVFSFETEGQLDDADSSSDQSLSESEPEMETRRRITSLPRPASLNAFDIIS-
MeCIPK14        IEFH--------------LEDSDLKRQE-------------------NHKSLNAFDIIS-
MeCIPK15        MKFQ--------------LEGFDMKVQENV----------------QNQKSLNAFDIIS-
MeCIPK11        IKFYDDY---------VGIDDTDKTDKQE-----------------PDVTNLNAFDLIS-
MeCIPK6         VR------------TKEEMEFDAFNG------EEDGNNGGKSKQ----PETLNAFHIIS-
MeCIPK7         IR------------SKEEMEFEAFNC------EEESKNGGKSKQ----PETLNAFHIIS-
MeCIPK18        SL----------IVETNREEHAFADCDAVFNVDESNSAVTQSKQESAKPCNLNAFDIIS-
MeCIPK22        SP----------MVETDTKGPAYLDCDAVN---ESSCAVTESKQE---LCYLNAFDIIS-
MeCIPK2         QK----------KTEAEVQDILDSNGSGPS---ENSSVSSEAKQESVKPPRLNAFDIIS-
MeCIPK13        AA----------ETKIDVKKLDPLVADTTLDPYENAFIFAEDKKELNRPISLNAFDIIS-
MeCIPK17        SV----------KAKTDGKNSEILVTDLSFHPSEESPSFSEDKKELGKPTSLNAFDIIS-
MeCIPK20        EAPPSPQGQARCTLLKDVHEAFNSSLPSENKNSSQKEVIAADTRSPVRPTNYNAFDIIS-
MeCIPK16        IT-----------ISNDLEEN-EKGRELIG---EEITKLSETNHS---PPFYNAFEFISA
MeCIPK25        LA-----------FSFQAISNPEKTEEEED---EALSAVMAKVSS---PKFFNAFEFISS
MeCIPK23        AFE---------QAE-VSLDDVNSIFNESGDCQNLVVEKRESPIGPVAPLTMNAFELIS-
MeCIPK5         IFE---------QSE-VILDNVSSIFNDTGDCHNLVVERQEAPIGPVAPITMNAFELIS-
MeCIPK10        QFE---------QGDDVNLDDVDAAFNDSK--EHLVTERKEK------PVSMNAFELIS-
MeCIPK9         QFQ---------QEDDVNLDDVDAAFDDSK--EHLVTERKGK------PVSMNAFELIS-
MeCIPK4         VFE---------EKEDTNLDDVEAVFKDSE--EHHVTEKKEE-----QPTAMNAFELIS-
MeCIPK3         VFE---------EKDDTNLDDVEAVFKDSK--DHLVTEKKEE-----HPAAMNAFELIS-
MeCIPK24        KHS---------EEGEVNLDDVRAVFDDIE--DQYVAEQSENSEG--GPLIMNAFEMIT-
MeCIPK8         RLI---------EYEDVNLDDVDAAFNDPE--EQKTDEQCGNEDT--GPLILNAFDMII-
MeCIPK21        FGY---------------ECDEKIYLDDVNAAFDVIEDDAAETKMPKPSSFINAFKLIA-
MeCIPK1         DPS---------------EEDDDIHID--NEAFSMQEVPLEGERSPGSPVLINAFQLIG-
                                                                    ***.:*
```

NAF/FISL motif

```
MeCIPK12        FSPGFDLSGLFEE---GGE-GARFVSGAPVSKIISKLEEIAKVVSFTVRT-KDYRVSLEG
MeCIPK19        FSPGFNLSGLFEE---GGEEGARIVSGAPVSKIISKLEEIAKLVSFTVRK-KDYRVSLEG
MeCIPK14        FSSGFDLSGLFNDCDISACN-ERFVSSESPAKIIDRIEEIAEAENVKVTKNRDCGAKLEG
MeCIPK15        FSSGFDLSCLFNECDVSASS-ERFVSCESPAKIIKRVEEIARTENIKLMKNKGWGAKLEG
MeCIPK11        FSSGLDLSGLFDDSYNAVEDGDRFVSSESPEKLVQKVEEFAKAERLRAKRKKEWAFEIEG
MeCIPK6         LSEGFDLSPLFEEKKRDEEEELRFATTRPASSVISRLEEVAKAGKFSVKKSE-TKVRMQG
MeCIPK7         LSEGFDLSPLFEEKKREEKEELRFATTRPASSVISRLEEVAKAGKFSVKKSE-TKVRMQG
MeCIPK18        YSAGFDLSGLFEE--KEKKKEVRFTANKPSSIIISKLEDIGKRLKLKMKKKDAGMLKFEG
MeCIPK22        YSAGFDLSGLFEE--KEKKKEMRFTAKKTASIIISKLEDTAKRLRLKIKKKDAGLLKFEG
MeCIPK2         LSAGFDLSGLFDE--NSQLREARFTSLQPASVIISKLEDVAKHLRLKIMKKEEGLLKMES
MeCIPK13        LSNGLDLSGFFTK--SQQEKETKFTSMHSASTIISKLEDTAKHLHLEVKKKDRGLLKLKG
MeCIPK17        LSDGFNLSGFFAK--SNKEKEAKFTSMHTASTIITKLEDTAKNLQLEVKQKDGGLLKLEG
MeCIPK20        RSKGFDLSGLFEED-RYQRLEARFTSKKPASTIVSKFQEIATMESFNFNKKD-GTVKLLG
MeCIPK16        MSSGFDLSSLFEN---KKKSGSMFTSKCAASVILTKLGSAAKKLNFRVLSDSEFKVKMQG
MeCIPK25        MSSGFDLSNLFEN---KKKTGSMFTSKFSASAIMNKIEAVAKGLNFKVSKVKDFKMRLQC
MeCIPK23        TSQGLNLSSLFEKQMGLVKRETRFTSKRSANEIISKIEEAATPLGFEVKKNN-FKMKLQG
MeCIPK5         TSQGLNLGSLFEKKMGLVKRETRFTSKHSANEIVSKIEEAAMPLGFEVKKNN-FKMKLQG
MeCIPK10        KTQGFSLDNLFEKQAGLVKRETRFASRTPANEIMSKIEDAAKPLGFNVDKRN-YKMKLKG
MeCIPK9         KTQGFSLENLFAKQAGIVKRETRFASHSPAKEIMSKIEEAAKPLGFNVDKRN-YKMKLKG
MeCIPK4         MSKGLNLGNLFDAEQGFKR-ETRFTSKRPANEIIHKIEEAAKPLGFDVHKKN-YKMRLEN
MeCIPK3         MSKGLNLGNLFDSEQ------------------VCMLEGLLY-----VVMLN-YIFKIDG
MeCIPK24        LSQGLNLSALFDRRQDYVKRQTRFVSRKPAKVIISTIEAVAESMSLKVHTRN-YKTRLEG
MeCIPK8         LSQGLNLATLFDRGKDSMKYQTRFISRKPARVVLSSMEVVAQSMGFKTHIRN-YKMRVEG
MeCIPK21        MSHDLDLSGLFQEQ-EDKKETTRLGSKHTVNETIKKIEAAALDLSLTVERMNNFRLKMHP
MeCIPK1         MSSCLDLSGFFEKE-DVSERKIRFTSNHSLKDLLEKIEDIARGMGFRIQKKN-GKLKVVQ
                 :  :.*   :*                    :  .                  .

MeCIPK12        SRE---GIKGPLTIAAEIFELTPKLVVVEVKKKGGDKGEYEEFCNKELKPGLQKLMQEES
MeCIPK19        SRE---GAKGPLTIAAEIFELTPKLVVVEVKKKGGDQGEYEEFCSKELKPGLQMLTQEEP
MeCIPK14        -------IDGTFVMAIEIYQLTEQLVVVEVKGKEMNAKPGQEIWKDKLRPKLGSL-VYEA
MeCIPK15        -------CDGSFAMAIEIYKLTDQLVVVEVKGKEMNAIPNQEIWKHKLRPQLDSL-VHKP
MeCIPK11        -------RNGNFGMEVEVYRLTENLAVVEARRRGGEAGCFKQMWKNKLKPELSGLTVSQP
MeCIPK6         QES---GRKGKLGIAAEIFAVTPSFLVVEVKKDNGDTLEYKQFCSKELRPALKDLVWTSP
MeCIPK7         QES---GRKGKLAIAAEIFAVTPSFLVVEVKKDNGDTLEYKQFCSKELRPALKDIVWTSP
MeCIPK18        SKA---GRKGALGIDAEIFEITPCFHLVEMKKSSGDTLEYQTVLNQEIRPALKDIVWTWQ
MeCIPK22        SNE---GRKGALGIDAQIFEITPYFHLVEMKKSSGDTIEYQALLKQEIRPALKDVIWTWQ
MeCIPK2         LQE---GRKGPLCIDAEIFEVAPNFHLVEVKKSNGDTMEYQKILKEDIKPALQDIVSAWQ
MeCIPK13        SEE---GRKGALSIDAEIFEFTPSFHMVEVKKSAGDTLEYTQILEQGLRPALKDIVWVWQ
MeCIPK17        SEE---GRKGALSIDAEIFEFTPSFHLVEVKKSAGDTLEYTQVLEQGIRPALKDIVWAWQ
MeCIPK20        CKE---GRKGQLEIDAEIFEVTPSFFVVELTKASGDTIEYKNFCNQELRPSLKDIVWAWQ
MeCIPK16        KEE---GRKGKLAVTAEVFEVAPEVAVVEFSKSAGDTLEYTKFCEEDVRPALKDIVWSWQ
MeCIPK25        PLE---GRKGRLSITAEVFEVALDVAVVEFSKSAGDTLEYAKFCDEDVRPALKDIVWTWQ
MeCIPK23        EKT---GRKGHLSIATEIFEVAPSLYMVELRKSGGDTLEFHKFY-KNLSAGLKDIVWRTI
MeCIPK5         EKT---GRKGQLSVATEIFEVAPSLHMVEVRKSCGDTLEFHKFY-KNLSVGLKDVVWKTI
MeCIPK10        DKN---GRKGQLSVATEVFEVAPSLHIVELRKIGGDTLEFHKFY-KTFSTGLKDVVWKSD
MeCIPK9         DKN---GRKGQLSVSTEVMS----------------------------------------
MeCIPK4         MKA---GRKGNLNVATEIFQVAPSLHMVEVRKAKGDTLEFHKFY-KNLSTCLDDVVWKSE
MeCIPK3         ALC---G-----------------------------------------CACA--------
MeCIPK24        ISA---NKAGQFAVVLEVFEVAPSLFMVDVRKASGDTLEYHKFY-KNFCAKLESIIWKPT
MeCIPK8         LSA---NKTAHFSVILEVFEVAPTFLMVDIQKAAGDASDYLKQS----------------
MeCIPK21        KQKMTRCTRSCFDLSAEVIEVAPTNCVVEISKSAGELRLFSEFC-KSLSTTLTEKSNTSF
MeCIPK1         ENKEQRSLRSLY-VAAEVFEISPSLHVVELQKSYGDASVYRQLC-KKLSNDLSVPSGQ--

MeCIPK12        ETADAASSQLPTEPLQLPTEPLQLPTESLQIPTEPLPIDTTHLPSDTE
MeCIPK19        EIVATASSQS-------------LPEHSHLFSAT----------SDTE
MeCIPK14        APTASGE-----------------------------------------
MeCIPK15        ATPAPSVTDYPISS----------------------------------
MeCIPK11        GTQVAGNC----------------------------------------
MeCIPK6         AGTSTIA-----------------------------------------
MeCIPK7         ADNSTIA-----------------------------------------
MeCIPK18        GEQQQQQQQQQILKQQQEQQELQPSHVCALQAASPYATS---------
MeCIPK22        GEQQQQ------LLKQEEQQEL--------------------------
MeCIPK2         GEQLLQ------PQQQQQEQETALAASAVMT-----------------
MeCIPK13        GEQQQKQ--------QQAHSS---------------------------
MeCIPK17        GEKQQQQE-----EQQQENSS---------------------------
MeCIPK20        GNDQQQS-----------------------------------------
MeCIPK16        GEDNCHQL-----ERTSGN-----------------------------
MeCIPK25        GDTVCSRN-----DSGENENQTSL------------------------
MeCIPK23        DEEKEEGQPEISGSSVVPA-----------------------------
MeCIPK5         DEEKEEEKVNSNGATVVPA-----------------------------
MeCIPK10        GT----------------------------------------------
MeCIPK9         ------------------------------------------------
MeCIPK4         DDMQEMK-----------------------------------------
MeCIPK3         ------------------------------------------------
MeCIPK24        DGVSASLLCGCEDKE---------------------------------
MeCIPK8         ------------------------------------------------
MeCIPK21        QVLDSEAPSIIDNNNQETIRSEKQIDREKNELGGYSSS----------
MeCIPK1         GLLTAQV-----------------------------------------
```
