# Supplementary figures and images for: Genome-wide gene phylogeny of CIPK family in cassava and expression analysis of partial drought-induced genes
Source: Front Plant Sci. 2015 Oct 30;6:914. doi: 10.3389/fpls.2015.00914 (PMC4626571; doi:10.3389/fpls.2015.00914)

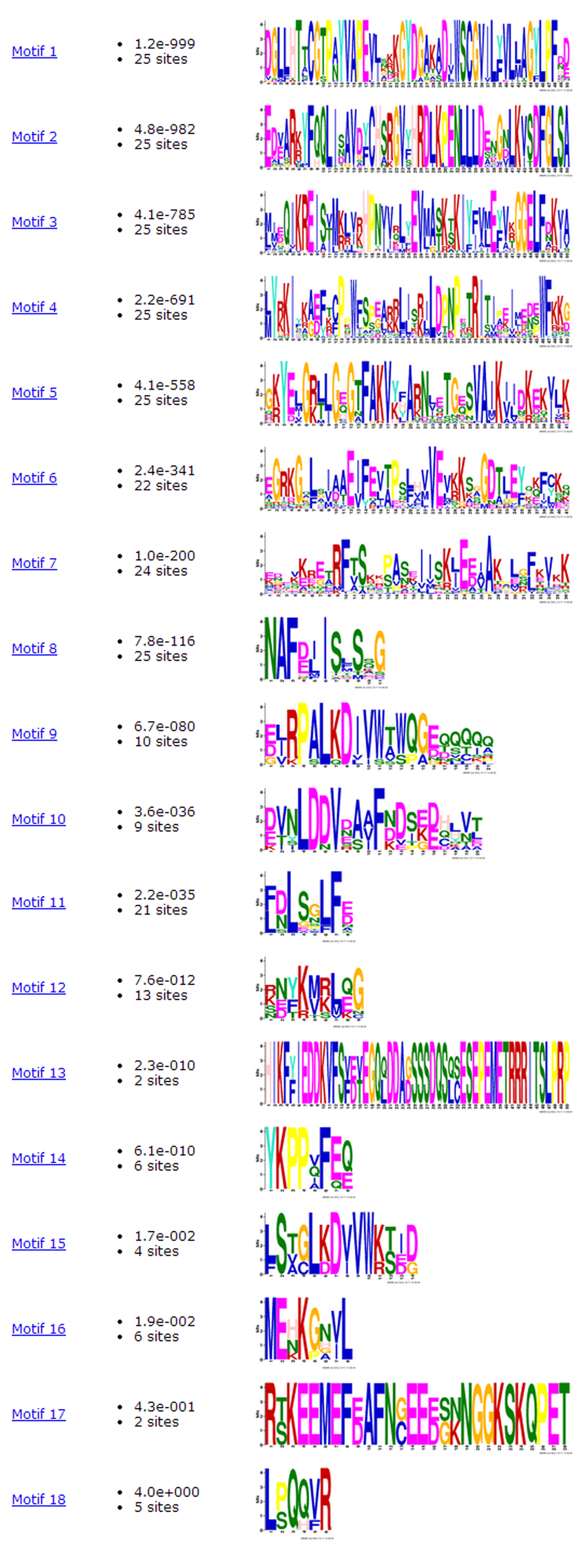

Supplement: Figure S2 — Motifs of CIPKs in cassava. [file Image2.TIF]

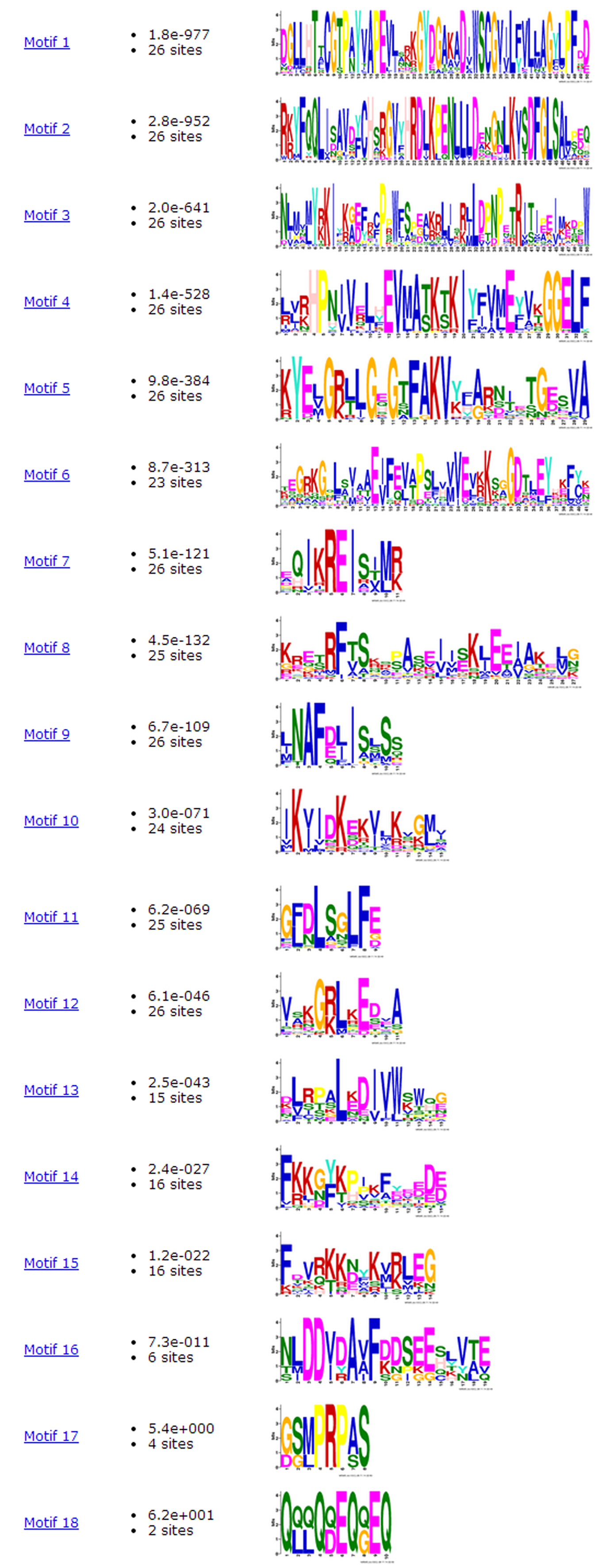

Supplement: Figure S3 — Motifs of CIPKs in Arabidopsis. [file Image3.TIF]

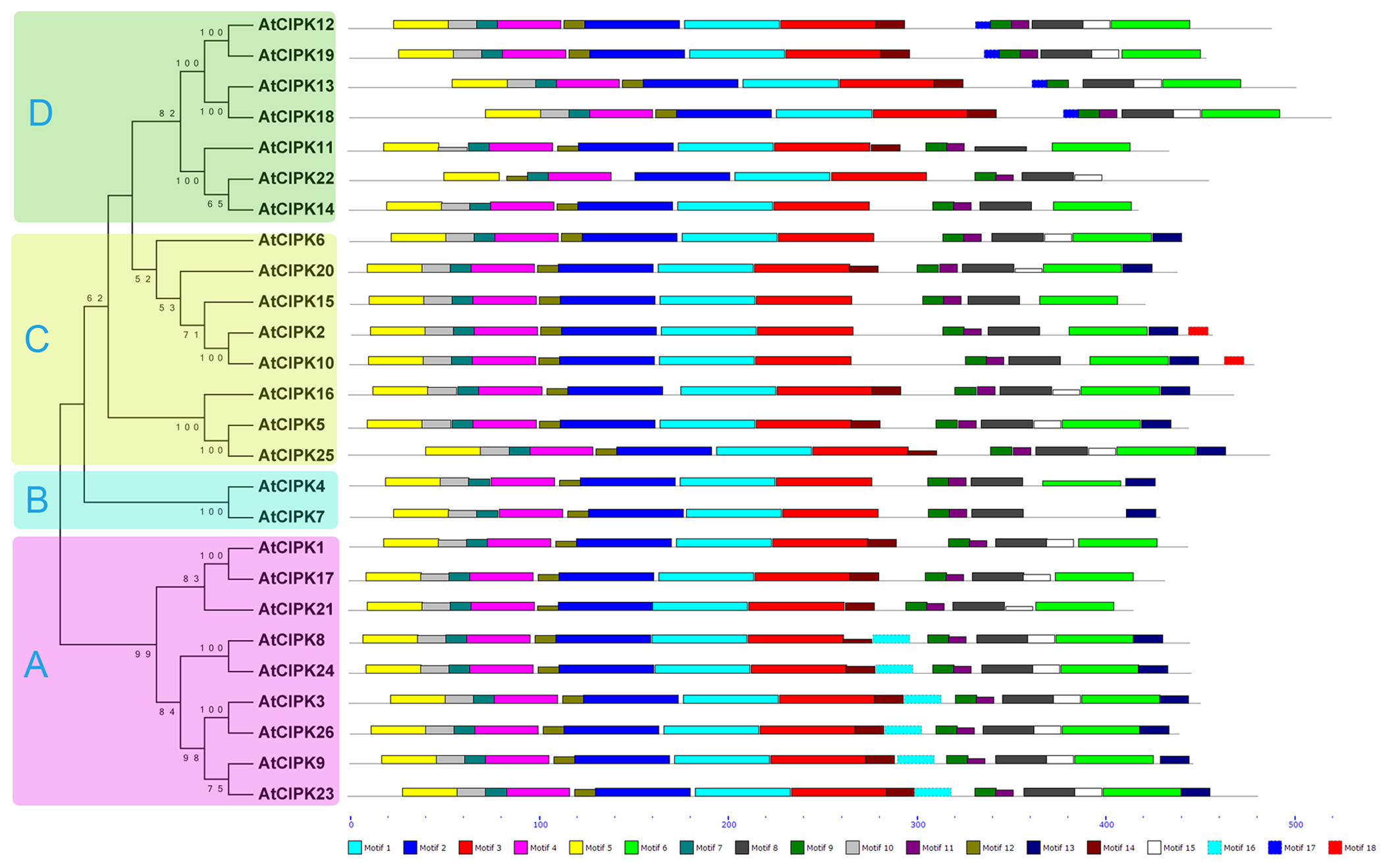

Supplement: Figure S4 — Conserved motifs of cassava CIPK proteins according to the phylogenetic relationship. [file Image4.TIF]

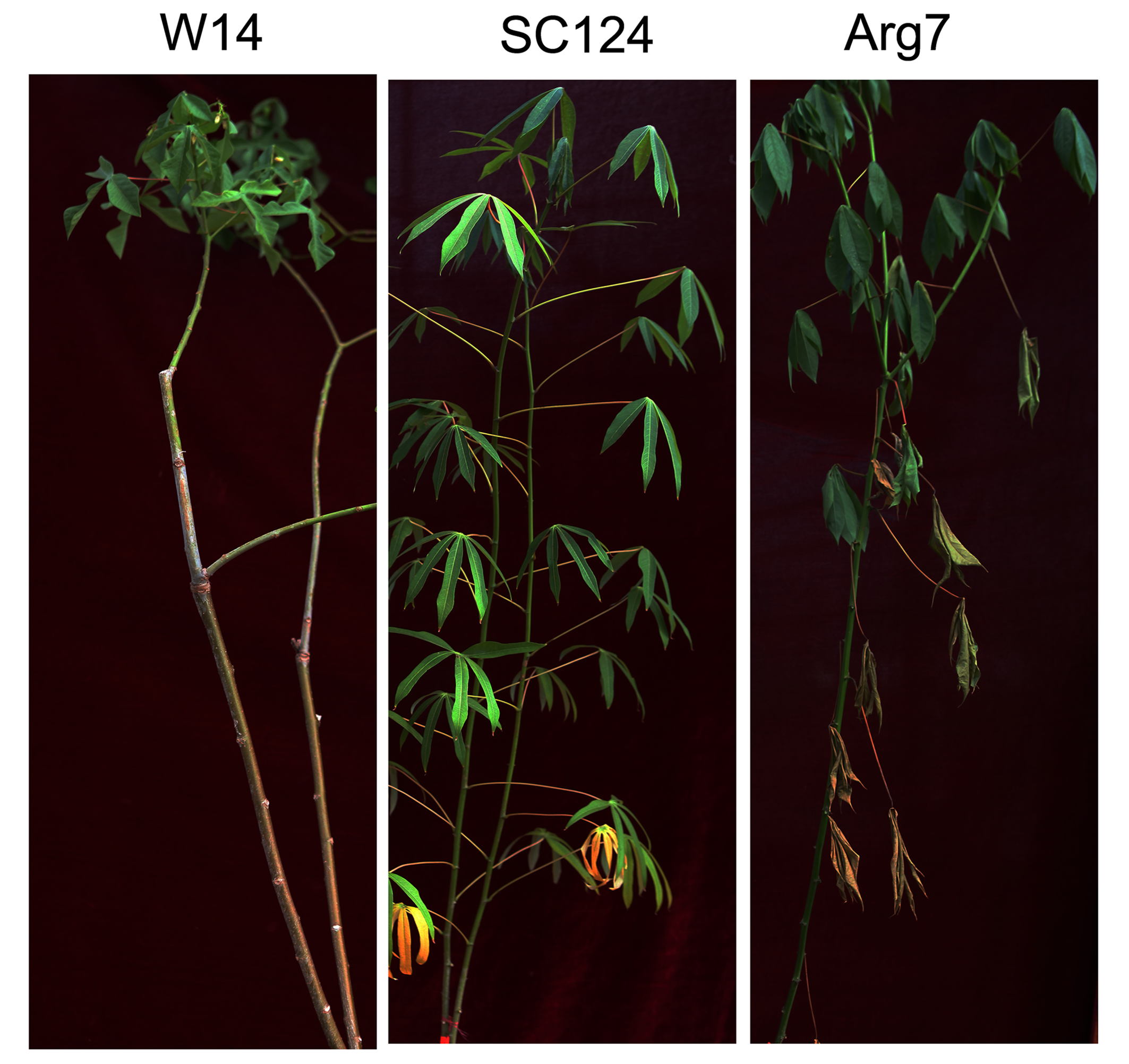

Supplement: Figure S5 — Photos of different accessions of cassava after 12 days drought treatment. [file Image5.TIF]

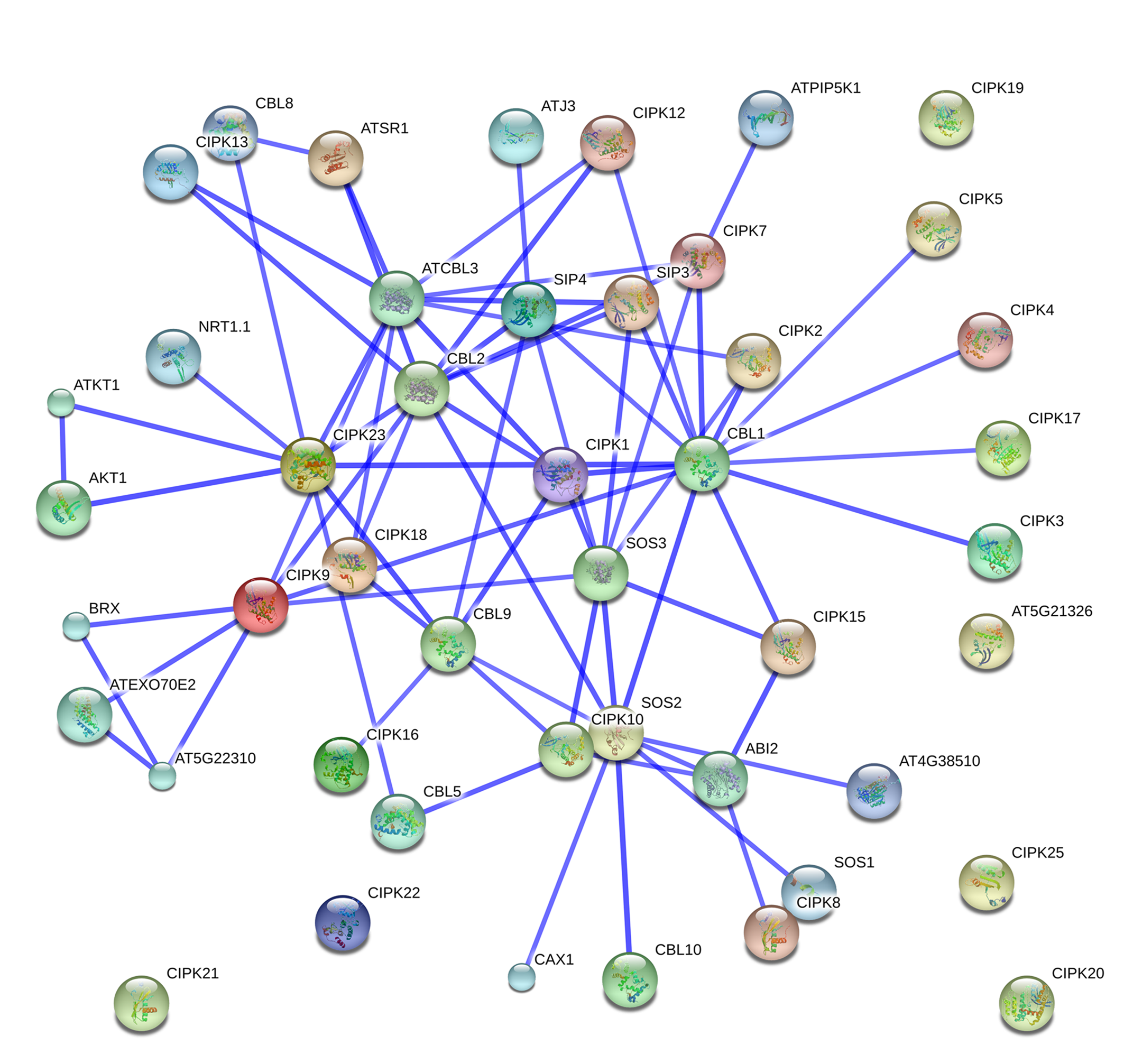

Supplement: Figure S6 — Interaction network of AtCIPKs by STRING. [file Image6.TIF]

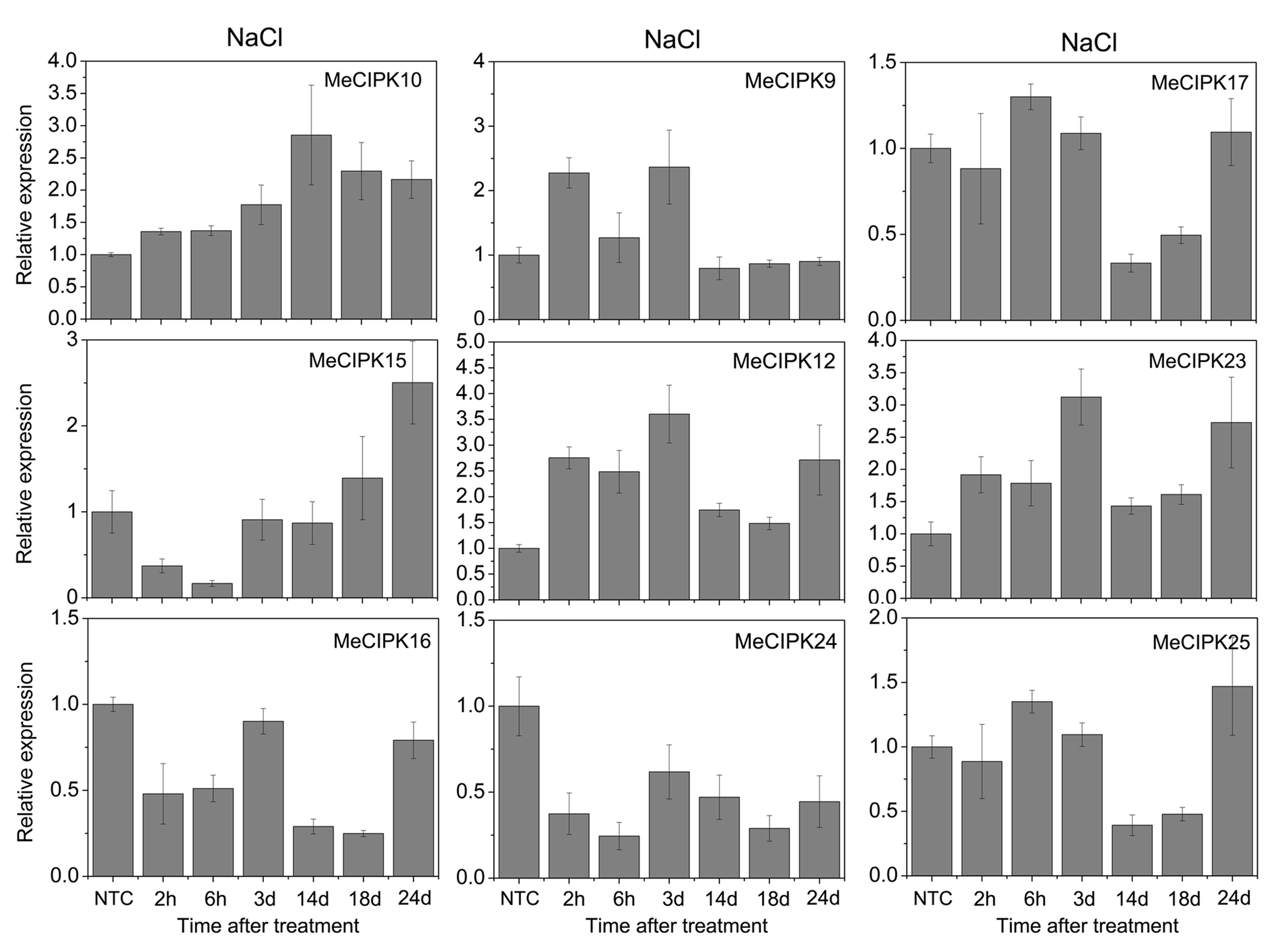

Supplement: Figure S7 — Expression profiles of CIPK genes in leaves of cassava in response to salt stress. The relative expression levels of each gene are presented as the mean fold changes between treated and control samples at each time point. NTC indicates no treatment controls (mean value = 1). Data are means ± SD of n = 3 biological replicates. [file Image7.TIF]

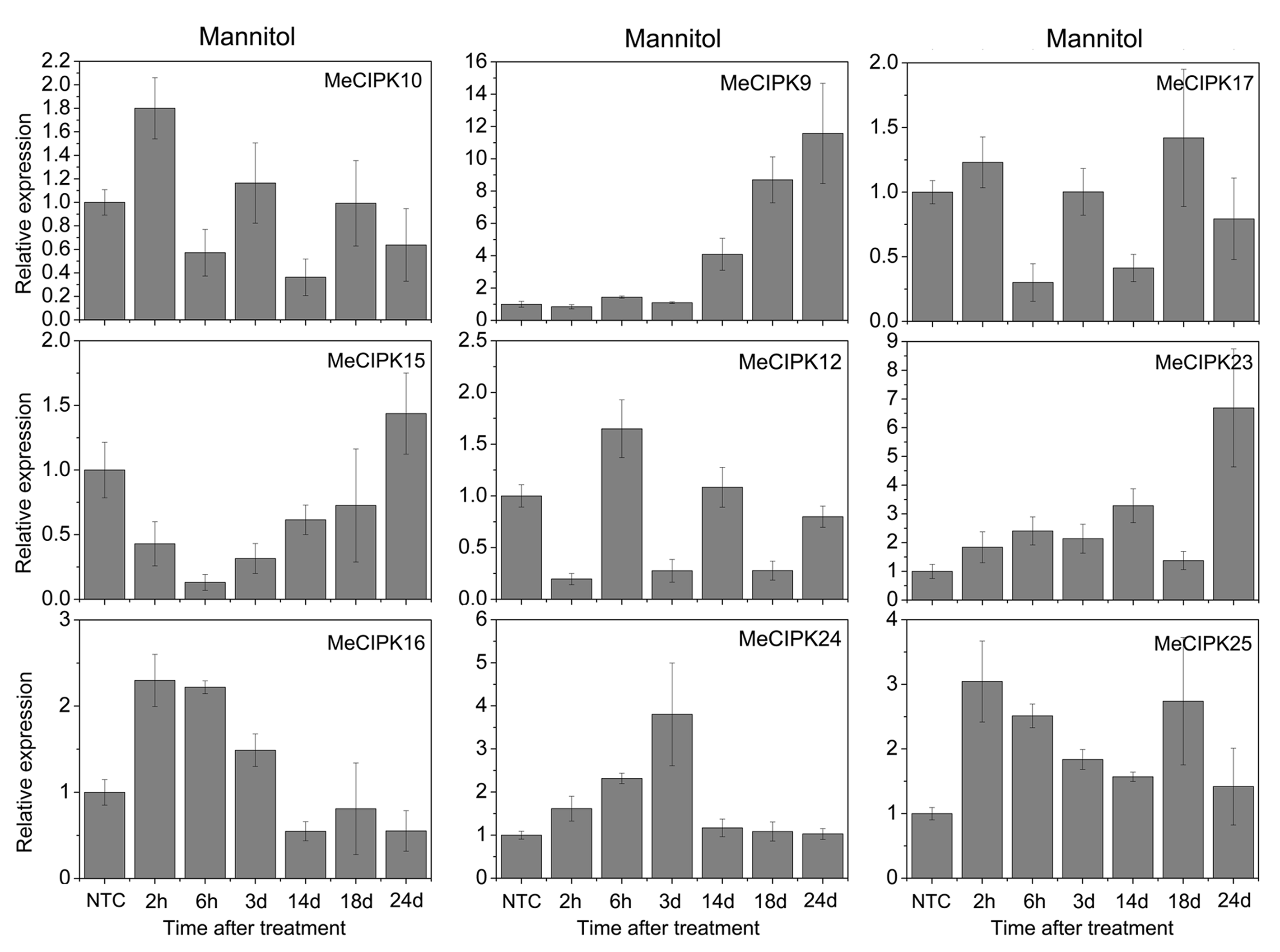

Supplement: Figure S8 — Expression profiles of CIPK genes in leaves of cassava in response to osmotic stress. The relative expression levels of each gene are presented as the mean fold changes between treated and control samples at each time point. NTC indicates no treatment controls (mean value = 1). Data are means ± SD of n = 3 biological replicates. [file Image8.TIF]

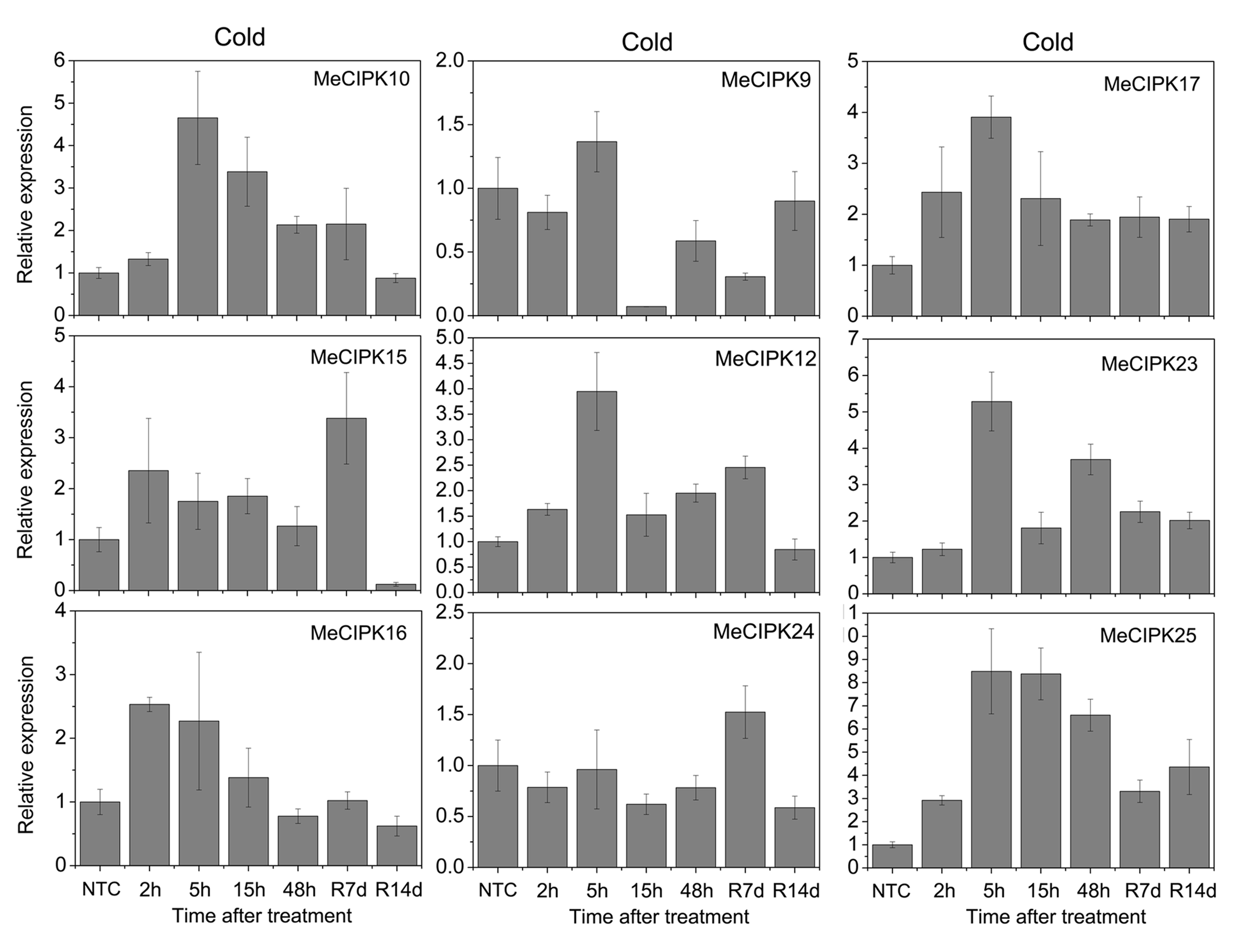

Supplement: Figure S9 — Expression profiles of CIPK genes in leaves of cassava in response to cold stress. R7d and R14d indicate 7 days and 14 days recovery, respectively. The relative expression levels of each gene are presented as the mean fold changes between treated and control samples at each time point. NTC indicates no treatment controls (mean value = 1). Data are means ± SD of n = 3 biological replicates. [file Image9.TIF]

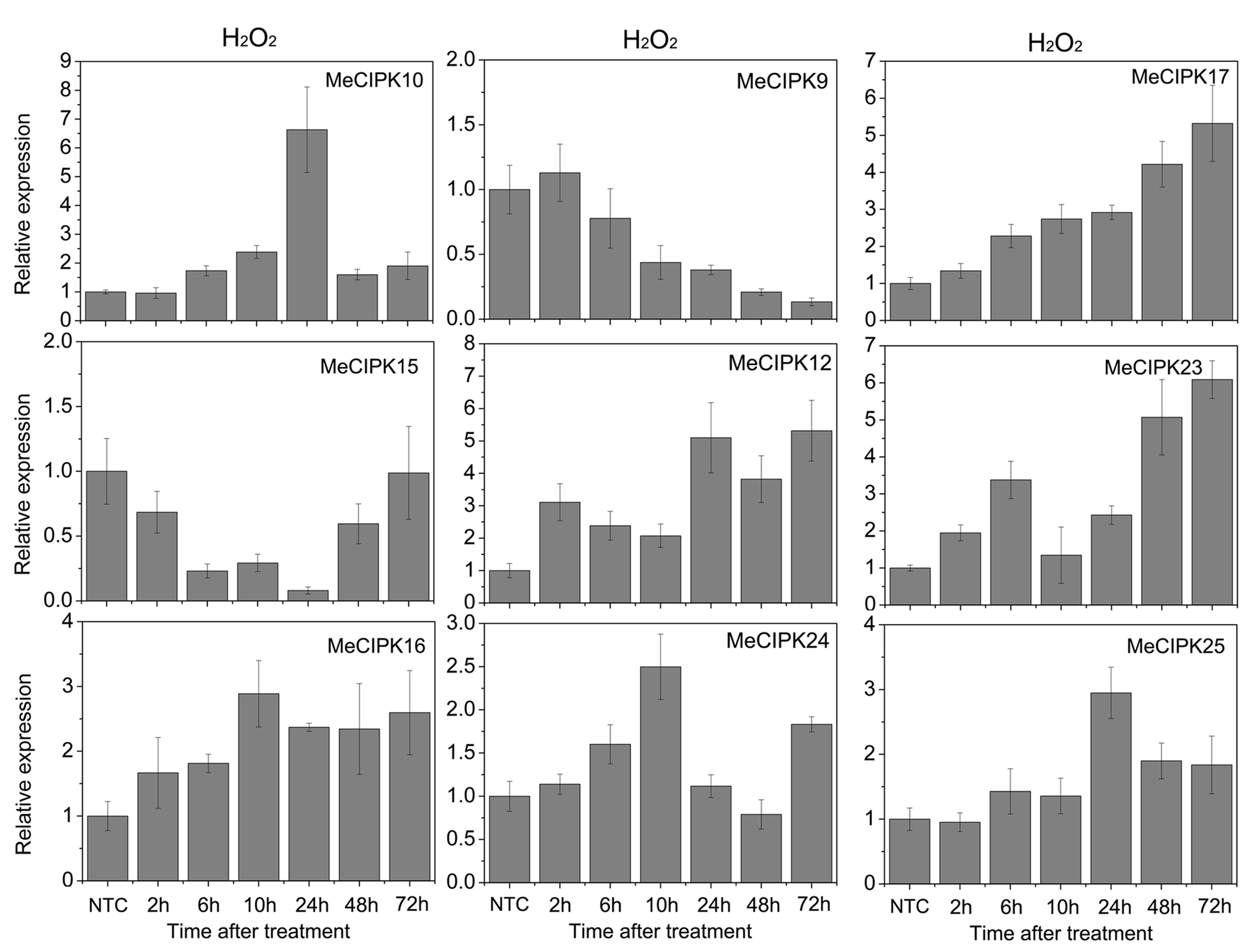

Supplement: Figure S10 — Expression profiles of CIPK genes in leaves of cassava in response to oxidative stress. The relative expression levels of each gene are presented as the mean fold changes between treated and control samples at each time point. NTC indicates no treatment controls (mean value = 1). Data are means ± SD of n = 3 biological replicates. [file Image10.TIF]

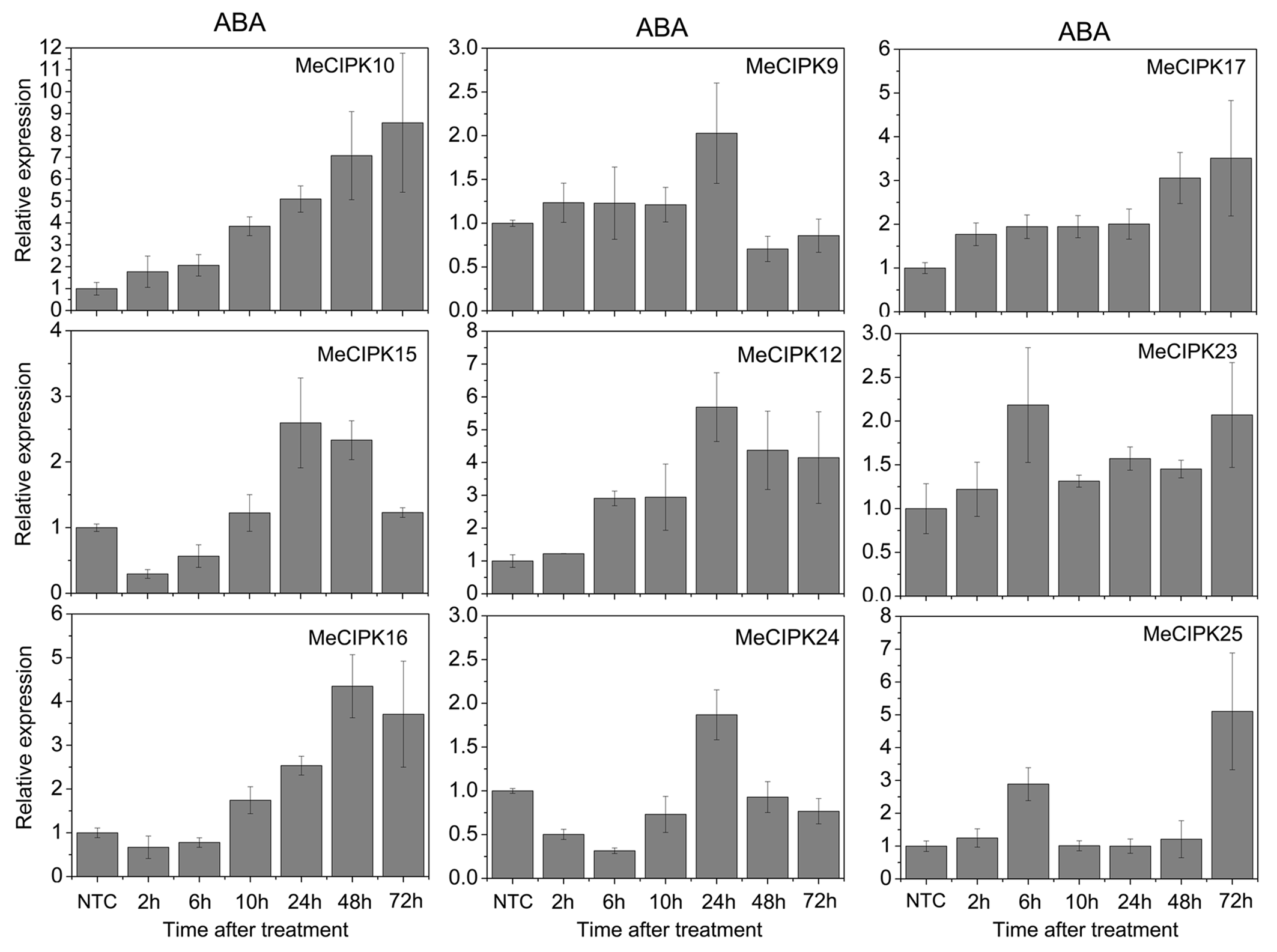

Supplement: Figure S11 — Expression profiles of CIPK genes in leaves of cassava in response to ABA. The relative expression levels of each gene are presented as the mean fold changes between treated and control samples at each time point. NTC indicates no treatment controls (mean value = 1). Data are means ± SD of n = 3 biological replicates. [file Image11.TIF]
